# Supplementary material for: Clinical characteristics, systemic complications, and in-hospital outcomes for patients with COVID-19 in Latin America. LIVEN-Covid-19 study: A prospective, multicenter, multinational, cohort study
Source: PLoS One. 2022 Mar 31;17(3):e0265529. doi: 10.1371/journal.pone.0265529 (PMC8970353; doi:10.1371/journal.pone.0265529)
Supplement: S1 Table — (DOCX) [file pone.0265529.s001.docx]

**ONLINE SUPPLEMENT**

**Title:** Clinical Characteristics, Systemic Complications, and In-Hospital Outcomes for Patients with COVID-19 in Latin America. LIVEN-Covid-19 Study: A Prospective, Multicenter, Multinational, Cohort Study.

**AUTHORS:** Luis F. Reyes^1, 2, 3*^, Alirio Bastidas^1^, Paula O. Narváez^1^, Daniela Parra-Tanoux^1^, Yuli V. Fuentes^1, 2^, Cristian C. Serrano-Mayorga^1, 2^, Valentina Ortíz^1^, Eder L. Caceres^1, 2^, Gustavo Ospina-Tascon^4, 5^, Ana M. Díaz^6^, Manuel Jibaja^6^, Magdalena Vera^7^, Edwin Silva^1, 8^, Luis Antonio Gorordo-Delsol^9^, Francesca Maraschin^3^, Fabio Varón-Vega^10^, Ricardo Buitrago^1, 8^, Marcela Poveda^1, 8^, Lina M. Saucedo^8^, Elisa Estenssoro^11^, Guillermo Ortíz^12^, Nicolás Nin^13^, Luis E. Calderón^4^, Gina S. Montaño^1^, Aldair J. Chaar^1^, Fernanda García^6^, Vanessa Ramírez^6^, Fabricio Picoita^6^, Cristian Peláez^6^, Luis Unigarro^6^, Gilberto Friedman^14^ Laura Cucunubo^10^, Alejandro Bruhn^7^, Glenn Hernández^7^ and Ignacio Martin-Loeches^15, 16^ for the LIVEN-Covid-19 Investigators^^^.

*^ The complete list of LIVEN-Covid-19 investigators is presented at the end of the manuscript.*

**AFFILIATIONS:**

1, Universidad de La Sabana, Chía, Colombia.

2, Department of Intensive Care, Clínica Universidad de La Sabana, Chía, Colombia.

3, Nuffield department of medicine, University of Oxford, Oxford, United Kingdom.

4, Department of Intensive Care, Fundación Valle del Lili, Cali, Colombia.

5, TransLab- CCM, Universidad Icesi, Cali, Colombia.

6, Critical Care Unit, Hospital Eugenio Espejo-Escuela de Medicina de la Universidad International, Quito, Ecuador.

7, Departamento de Medicina Intensiva, Facultad de Medicina, Pontificia Universidad Católica de Chile, Santiago, Chile.

8, Fundación Clínica Shaio, Bogota, Colombia.

9, Critical Care Unit, Hospital Juárez de México, Ciudad de México, México.

10, Department of Intensive Care, Fundación Neumológica Colombiana-Fundación Cardioinfantil, Bogotá, Colombia.

11, Hospital Interzonal de Agudos San Martín de La Plata, La Plata, Argentina.

12, Universidad del Bosque, Bogotá, Colombia.

13, Intensive Care Unit, Hospital Español, Montevideo, Uruguay.

14, School of Medicine, Universidad de Federal do Rio Grande do Sul, Porto Alegre, Brazil,

15, Department of Clinical Medicine, St James's Hospital, Multidisciplinary Intensive Care Research Organization (MICRO), Dublin, Ireland.

16, Hospital Clinic, IDIBAPS, Universidad de Barcelona, CIBERes, Barcelona, Spain.

***Corresponding author:** Luis Felipe Reyes, MD., PhD., Universidad de La Sabana, Chía, Colombia. Phone number: 57-1 861 55 55 Ext: 23342. Email: [luis.reyes5@unisabana.edu.co](mailto:luis.reyes5@unisabana.edu.co)

**Abbreviated running title:** Severe COVID-19 in Latin America

**SUPLEMENTAL TABLE 1**

| **Supplement Table 1.** Treatments stratified by patients admitted to the Intensive Care Unit (ICU). | | | | |
| --- | --- | --- | --- | --- |
|  |  | **Patients admitted to the ICU** | |  |
| **Treatment** | **All**  n=3008 | **No**  n=1074 | **Yes**  n=1934 | ***p*** *value* |
| Antibiotic | 1456 (48.4) | 273 (25.4) | 1183 (61.2) | **<0.001** |
| Neuraminidase inhibitors | 82 (2.7) | 6 (0.6) | 76 (3.9) | **<0.001** |
| Dialysis/hemofiltration | 346 (11.5) | 9 (0.8) | 337 (17.4) | **<0.001** |
| Vasopressors or inotropes | 1111 (36.9) | 11 (1) | 1100 (57) | **<0.001** |
| Corticosteroids | 1578 (52.5) | 244 (22.7) | 1334 (69) | **<0.001** |
| Hydrocortisone | 155 (5.2) | 4 (0.4) | 151 (7.8) | **<0.001** |
| Methylprednisolone | 545 (18.1) | 41 (3.8) | 504 (26.1) | **<0.001** |
| Dexamethasone | 1145 (38.1) | 193 (18) | 952 (49.2) | **<0.001** |
| ECMO | 36 (1.2) | 0 | 36 (1.9) | **<0.001** |
| Lopinavir/ritonavir | 149 (5) | 37 (3.4) | 112 (5.8) | **<0.001** |
| Ribavirin | 1 (0) | 1 (0.1) | 0 (0) | 0.180 |
| Remdesivir | 24 (0.8) | 0 (0) | 24 (1.2) | **<0.001** |
| Invasive Mechanical Ventilation | 1407 (46.7) | 16 (1.5) | 1391 (71.9) | **<0.001** |
| Tracheostomy inserted | 298 (9.9) | 5 (0.5) | 293 (15) | **<0.001** |
| Thoracostomy | 34 (1.1) | 5 (0.5) | 29 (1.5) | **<0.001** |
| Antifungal | 3200 (7.9) | 2384 (11.9) | 816 (4.0) | **<0.001** |
| Angiotensin II Receptor Blockers | 2865 (7.1) | 1674 (8.4) | 1191 (5.8) | **<0.001** |
| Therapeutic anticoagulant | 532 (1.3) | 510 (2.5) | 22 (0.1) | **<0.001** |
| Chloroquine | 89 (3) | 17 (1.6) | 72 (3.7) | **<0.001** |
| Hydroxychloroquine | 91 (3) | 23 (2.1) | 68 (3.5) | 0.035 |
| Convalescent plasma | 32 (1.1) | 0 (0) | 32 (1.7) | **<0.001** |
| Macrolides | 2 (0.1) | 1 (0.1) | 1 (0.1) | 0.673 |
| Interferon alfa | 1 (0) | 1 (0.1) | 0 | 0.180 |
| Interferon beta | 3 (0.1) | 1 (0.1) | 2 (0.1) | 0.932 |
| Ivermectina | 14 (0.5) | 9 (0.8) | 5 (0.3) | 0.025 |
| Colchicine | 24 (0.8) | 10 (0.9) | 14 (0.7) | 0.541 |
| Melatonina | 20 (0.7) | 12 (1.1) | 8 (0.4) | 0.023 |
| Tocilizumab | 30 (1) | 0 (0) | 31 (1.6) | **<0.001** |
| Vitamin C | 12 (0.4) | 5 (0.5) | 7 (0.4) | 0.66 |
| **ECMO,** Extracorporeal membrane oxygenation. | | | | |

| **Supplement Table 2.** Patients with severe COVID-19 that developed complications stratified by patients admitted to the Intensive Care Unit (ICU). | | | | | |
| --- | --- | --- | --- | --- | --- |
|  |  | **Patients admitted to the ICU** | |  |  |
| **Complications** | **All**  n=3008 | **No**  n=1074 | **Yes**  n=1934 | *p* *value* |  |
| ***Neurologic, n (%)*** | | | | | |
| Seizures | 22 (0.7) | 5 (0.5) | 17 (0.9) | 0.202 |  |
| Stroke | 38 (1.3) | 4 (0.4) | 34 (1.8) | 0.001 |  |
| Meningitis or Encephalitis | 7 (0.2) | 2 (0.2) | 5 (0.3) | 0.693 |  |
| ***Cardiovascular, n (%)*** | | | | | |
| Congestive Heart Failure | 143 (4.8) | 14 (1.3) | 129 (6.7) | **<0.001** |  |
| Endocarditis, Myocarditis, Pericarditis | 89 (3) | 8 (0.7) | 81 (4.2) | **<0.001** |  |
| Cardiac Arrhythmia | 225 (7.5) | 10 (0.9) | 215 (11.1) | **<0.001** |  |
| Cardiac Ischemia | 86 (2.9) | 8 (0.7) | 78 (4) | **<0.001** |  |
| Myocardial Infarction | 55 (1.8) | 6 (0.6) | 49 (2.5) | **<0.001** |  |
| ***Pulmonary, n (%)*** | | | | | |
| Acute Respiratory Distress Syndrome | 1179 (56.2) | 92 (8.6) | 1087 (56.2) | **<0.001** |  |
| Pneumothorax | 45 (1.5) | 4 (0.4) | 41 (2.1) | **<0.001** |  |
| Pleural Effusion | 116 (3.9) | 26 (2.4) | 90 (4.7) | **<0.001** |  |
| Pulmonary Embolism | 272 (0.7) | 237 (1.2) | 35 (0.2) | **<0.001** |  |
| Cryptogenic Organizing Pneumonia | 17 (0.6) | 7 (0.7) | 10 (0.5) | 0.637 |  |
| ***Gastrointestinal, n (%)*** | | | | | |
| Pancreatitis | 6 (0.2) | 2 (0.2) | 4 (0.2) | 0.903 |  |
| Liver Dysfunction | 128 (4.3) | 4 (0.4) | 124 (6.4) | **<0.001** |  |
| Gastrointestinal Hemorrhage | 56 (1.9) | 3 (0.3) | 53 (2.7) | **<0.001** |  |
| ***Renal, n (%)*** | | | | | |
| Acute kidney Injury | 692 (23) | 31 (2.9) | 661 (34.2) | **<0.001** |  |
| ***Metabolic, n (%)*** | | | | | |
| Hyperglycemia | 527 (17.5) | 42 (3.9) | 485 (25) | **<0.001** |  |
| Hypoglycemia | 60 (2) | 13 (1.2) | 47 (2.4) | 0.022 |  |
| ***Hematologic, n (%)*** | | | | | |
| Anemia | 476 (15.8) | 11 (1) | 465 (24) | **<0.001** |  |
| Disseminated Intravascular Coagulation | 129 (4.3) | 8 (0.7) | 120 (6.2) | **<0.001** |  |
| Bleeding | 84 (0.2) | 80 (0.4) | 4 (0.0) | **<0.001** |  |
