# Supplementary material for: Clinical characteristics, systemic complications, and in-hospital outcomes for patients with COVID-19 in Latin America. LIVEN-Covid-19 study: A prospective, multicenter, multinational, cohort study
Source: PLoS One. 2022 Mar 31;17(3):e0265529. doi: 10.1371/journal.pone.0265529 (PMC8970353; doi:10.1371/journal.pone.0265529)
Supplement: S1 File — (PDF) [file pone.0265529.s003.pdf]

CRF Completion Guide

DESIGN OF THIS CASE REPORT FORM (CRF)

This CRF is set up in modules to be used for recording data on the ISARIC_nCov Core Database or for independent studies.

Module 1 and Module 2 complete on the first day of admission or on first day of COVID-19 assessment.
Module 2 also complete on first day of admission to ICU or high dependency unit. In addition, complete daily for as many days as resources allow up to a maximum of 14 days. Continue to follow-up patients who transfer between wards.
Module 3 (Outcome) complete at discharge or death

GENERAL GUIDANCE

- The CRF is designed to collect data obtained through examination, interview and review of hospital notes. Data may be collected prospectively or retrospectively if the patient is enrolled after the admission date.
- Participant Identification Numbers consist of a 5 digit site code and a 4 digit participant number. You can obtain a site code and registering on the data management system by contacting *ncov@isaric.org*. Participant numbers should be assigned sequentially for each site beginning with 0001. In the case of a single site recruiting participants on different wards, or where it is otherwise difficult to assign sequential numbers, it is acceptable to assign numbers in blocks or incorporating alpha characters. E.g. Ward X will assign numbers from 0001 or A001 onwards and Ward Y will assign numbers from 5001 or B001 onwards. Enter the Participant Identification Number at the top of every page.
- Printed paper CRFs may be used for later transfer of the data onto the electronic database.
- In the case of a participant transferring between sites, it is preferred to maintain the same Participant Identification Number (PIN) across the sites. When this is not possible, the first site should record 'Transfer to other facility' as an OUTCOME and the second site should start a new form with a new PIN and indicate 'YES-transferred' in the RE-ADMISSION section. If the PIN from the previous site is eventually obtained this can be entered under 'If YES 'Participant Identification Number:'
- For participants who are re-admitted with COVID-19 to the same site, **start a new form with a different Participant Identification Number (PIN)** and enter the previous PIN in response to the question 'Previous participant ID'.
- Complete every line of every section, except where the instructions say to skip a section based on a response.
- Selections with circles (**O**) are single selection answers (choose one answer only). Selections with square boxes (☐) are multiple selection answers (choose as many answers as are applicable).
- Mark 'Not done' for any results of laboratory values that are not available, not applicable or unknown.
- Avoid recording data outside of the dedicated areas. Sections are available for recording additional information.
- If using paper CRFs, we recommend writing clearly in ink, using BLOCK-CAPITAL LETTERS.
- Place an (X) when you choose the corresponding answer. To make corrections, strike through (-------) the data you wish to delete and write the correct data above it. Please initial and date all corrections.
- Please keep all of the sheets for a single participant together e.g. with a staple or participant-unique folder.
- Please transfer all paper CRF data to the electronic database. All paper CRFs needs to be stored locally, do not send any forms to us. Data are accepted only via secure electronic database.
- Please enter data on the electronic data capture system at https://ncov.medsci.ox.ac.uk/. If your site would like to collect data independently, we are happy to support the establishment of locally hosted databases.
- Please contact us at *ncov@isaric.org* if you need help with databases, if you have comments and to let us know that you are using the forms.

CRF Completion Guide

FURTHER GUIDANCE AND DEFINITIONS

Comorbidities

Comorbidities present before the onset of COVID-19 and are still present. Do not include those that developed following the onset of COVID-19 symptoms. More detailed guidance is provided.

Hospital admission

For patients who were admitted to hospital with COVID-19 or symptoms consistent with possible COVID-19 infection, please enter details for the date of hospital admission. For patients with a clear alternative diagnosis leading to admission who subsequently acquired COVID-19, original admission date should be provided, but all subsequent references to admission should be taken as referring to day COVID-19 was first clinically suspected (or within the first 24 hours after first day of suspected or confirmed COVID-19 infection).

Where a patient was admitted via multiple hospital departments, count admission from the time they came to the first department during the visit that led to their admission (e.g. arrival at the Emergency Department).

Oxygen therapy

Include any form of supplemental oxygen received using any methods.

Invasive ventilation

Please include any mechanical ventilation delivered following intubation or via a tracheostomy. Do not include patients who are breathing independently via a tracheostomy.

Non-invasive ventilation

Please include any positive-pressure treatment given via a tight-fitted mask. This can be continuous positive pressure (CPAP) or bi-level positive pressure (BIPAP).

Renal replacement therapy or dialysis

Please include any form of continuous renal replacement therapy or intermittent haemodialysis.

Worst result

References to 'worst result' refer to those furthest from the normal physiological range or laboratory normal range.

Results that were rejected by the clinical team (e.g. pulse oximetry on poorly perfused extremities, haemolysed blood samples, contaminated microbiology results) should not be reported.

The following measures should be considered as a single observation and entered together:

Blood gas results: Please report the measures from the blood gas with the lowest pH (most acidotic).

Blood pressure: Please report the systolic and diastolic blood pressure from the observation with the lowest mean arterial pressure (if mean arterial pressure has not been calculated, report the measurement with lowest systolic blood pressure).

Respiratory rate: If both abnormal low and high rate observed, record the abnormally high rate.

MODULE 1: PRESENTATION/ADMISSION CASE REPORT FORM

CLINICAL INCLUSION CRITERIA

Suspected or confirmed novel coronavirus (COVID-19) infection:

Select yes if patient has either clinically suspected or laboratory-confirmed SARS-CoV-2 /COVID-19 infection.

DEMOGRAPHICS

Enrolment date: Date of enrolment into the study or for in-patients is the date that COVID-19 was first assessed as suspected or confirmed by a clinician.

Ethnic group:

Please enter all that apply of the following choices which best describe the patient's ethnicity or major ethnic group at birth. Please exclude nationality as nations often include many different ethnic groups (For example, Singaporean is the nationality but the ethnic grouping within Singapore could be East Asian, South Asian etc.) Cross (X) all that apply. If 'Other' write the full name of the ethnic group of the patient. Please do not enter a letter or number corresponding to a local/national ethnicity coding system.

If the patient's ethnicity is not known, please place a cross (X) in the 'Unknown' box.

Post-partum: Defined as within six weeks of delivery.

If the baby is positive for COVID-19 please complete a separate form for the baby as well.

ONSET & ADMISSION

Onset date of first/earliest symptom: Please provide the date of patient reported onset of the first symptom that you clinically believe was related to this episode of COVID-19 infection.

Most recent presentation/admission date at this facility:

Where a patient was admitted via multiple hospital departments, count admission from the time they came to the first department during the visit that led to their admission (e.g. arrival at the Emergency Department). For patients with a clear alternative diagnosis leading to admission who subsequently acquired COVID-19 report the date of admission as the day they were admitted to the healthcare facility.

RE-ADMISSION

Was the patient admitted previously or transferred from any other facility during this illness episode?

For participants who return for re-admission to the same site, start a new form with the same Participant Identification Number. Please check "YES-admitted previously to this facility". Enter each re-admission as a separate entry in the electronic database.

For participants who transfer between two sites that are both collecting data on this form, it is preferred to have the data entered by a single site as a single admission, under the same Participant Identification Number. When this is not possible, the first site should record "Transfer to other facility" as an OUTCOME, and the second site should start a new form with a new patient number and indicate "YES-transferred from other facility" in RE- ADMISSION.

For participants who return for re-admission to the same site, **start a new form with a different Participant Identification Number**. Please check "YES-admitted previously to this facility" in the RE-ADMISSION section. Enter as a separate entry in the electronic database.

MODULE 1: PRESENTATION/ADMISSION CASE REPORT FORM

CLINICAL INCLUSION CRITERIA

Suspected or confirmed novel coronavirus (COVID-19) infection: OYES ONO

DEMOGRAPHICS

Clinical centre name:____________________ Country:________________

Enrolment date /first COVID-19 assessment date: [_D_][_D_]/[_M_][_M_]/[_2_][_0_][_Y_][_Y_]

Ethnic group *(check all that apply)*: ☐Arab ☐Black ☐East Asian ☐South Asian ☐ West Asian ☐Latin American ☐White ☐Aboriginal/First Nations ☐Other: ____________ OUnknown

Employed as a Healthcare Worker? OYES ONO OUnknown Employed in a microbiology laboratory? OYES ONO OUnknown

Sex at Birth: OMale OFemale ONot specified/Unknown Age [___][___][___]years OR [___][___]months

Pregnant? OYES ONO OUnknown If YES: Gestational weeks assessment: [___][___] weeks

POST PARTUM (within 6 weeks of delivery)? OYES ONO OUnknown *(if NO or Unknown skip this section)*

Pregnancy Outcome: OLive birth OStill birth Delivery date: [_D_][_D_]/[_M_][_M_]/[_2_][_0_][_Y_][_Y_]

Baby tested for COVID-19/SARS-CoV-2 infection? OYES ONO OUnknown

If YES, result of test: OPositive ONegative OUnknown (If Positive, complete a separate CRF for baby)

INFANT – Less than 1 year old? OYES ONO *(If NO skip this section)*

Birth weight: [___][___].[___]Okg or Olbs OUnknown

Gestational outcome: O Term birth (≥37wk GA) OPreterm birth (<37wk GA) OUnknown

Breastfed? OYES-currently breastfeeding OYES-breastfeeding discontinued ONO OUnknown

Vaccinations appropriate for age/country? OYES ONO OUnknown

ONSET & ADMISSION

Onset date of first/earliest symptom: [_D_][_D_]/[_M_][_M_]/[_2_][_0_][_Y_][_Y_]

Most recent presentation/admission date at this facility: [_D_][_D_]/[_M_][_M_]/[_2_][_0_][_Y_][_Y_]

RE-ADMISSION

Was the patient admitted previously or transferred from any other facility during this illness episode?

OYES-admitted previously to this facility OYES–transferred from other facility ONO OUnknown

Has this patient's data been previously collected under a different patient number? OYES ONO OUnknown

If YES, Participant Identification number (PIN): ____________

Is the patient being re-admitted with or due to COVID-19? *(Please only add re-admission episodes for COVID related complications or patients remaining positive). Assign new subject ID* OYES ONO OUnknown

Previous participant ID: ____________ OUnknown

Number of re-admissions: _____ (record as a new patient for each re-admission)

Please provide reason for readmission: ____________________

SIGNS AND SYMPTOMS AT HOSPITAL ADMISSION

Please provide details of clinical observations made as close to presentation/admission, or within 24 hours of admission. For observations not made immediately at admission, please record the first available data (patient reported and/or from medical records) within 24 hours of admission. For patients with a clear alternative diagnosis leading to admission who subsequently acquired COVID-19, complete these observations for the 24 hours after onset of symptoms of suspected or confirmed COVID-19 infection.

Temperature

Please enter the peripheral body temperature (rectal if < 3 months) in the space provided and indicate the unit of measurement, either degrees Celsius (°C) or Fahrenheit (°F).

Heart rate (HR)

Enter the heart rate measured in beats per minute. This may be measured manually or by electronic monitoring.

Respiratory rate (RR)

Enter the respiratory rate in breaths per minute. Manual rather than electronic measurement is preferred where possible (this is achieved by counting the number of breaths for one minute, counting how many times the chest rises within this time period). Record the highest respiratory rate documented on admission.

Systolic BP

Please enter the systolic blood pressure measured in millimetres of mercury (mmHg), in the relevant sections. For example, if the blood pressure is 120/85 mmHg, enter 120 in the section marked 'systolic BP'. Use any recognised method for measuring blood pressure.

Diastolic BP

Please enter the diastolic blood pressure measured in millimetres of mercury (mmHg), in the relevant sections. For example, if the blood pressure is 120/85 mmHg, enter 85 in the section marked 'diastolic BP'. Use any recognised method for measuring blood pressure.

Oxygen saturation

For all patients, irrespective of ventilation or supplemental oxygen requirement, please enter the percentage oxygen saturation (the percentage of haemoglobin binding sites in the bloodstream occupied by oxygen) at the time of admission. This may be measured by pulse oximetry or by arterial blood gas analysis.

Sternal capillary refill time > 2 seconds?

Sternal capillary refill time is measured by pressing on the sternum for five seconds with a finger or thumb until the underlying skin turns white and then noting the time in seconds needed for the colour to return once the pressure is released.

MODULE 1: PRESENTATION/ADMISSION CASE REPORT FORM

SIGNS AND SYMPTOMS AT HOSPITAL ADMISSION *(first available data at presentation/admission – within 24 hours)*

Temperature: [___][___][___].[___]O°C *or* O°F

HR: [___][___][___]beats/minute **RR:** [___][___]breaths/minute

Systolic BP: [___][___][___]mmHg **Diastolic BP:** [___][___][___]mmHg

Oxygen saturation: [___][___][___]% **On:** ORoom air OOxygen therapy OUnknown

Sternal capillary refill time >2sec. OYES ONO OUnknown **Height:** [___][___][___]cm **Weight:** [___][___][___]kg

SIGNS AND SYMPTOMS ON ADMISSION *(Unk = Unknown)*			
History of fever	OYES ONO OUnk	Fatigue / Malaise	OYES ONO OUnk
Cough OYES - non-productive OYES - productive		Anorexia	OYES ONO OUnk
OYES - with haemoptysis	ONO OUnk	Altered consciousness/confusion	OYES ONO OUnk
Sore throat	OYES ONO OUnk	Muscle aches (myalgia)	OYES ONO OUnk
Runny nose (rhinorrhoea)	OYES ONO OUnk	Joint pain (arthralgia)	OYES ONO OUnk
Wheezing	OYES ONO OUnk	Inability to walk	OYES ONO OUnk
Shortness of breath	OYES ONO OUnk	Abdominal pain	OYES ONO OUnk
Lower chest wall indrawing	OYES ONO OUnk	Diarrhoea	OYES ONO OUnk
Chest pain	OYES ONO OUnk	Vomiting / Nausea	OYES ONO OUnk
Conjunctivitis	OYES ONO OUnk	Skin rash	OYES ONO OUnk
Lymphadenopathy	OYES ONO OUnk	Bleeding (Haemorrhage)	OYES ONO OUnk
Headache	OYES ONO OUnk	If YES, specify site(s):________________	
Loss of smell (Anosmia)	OYES ONO OUnk	Other symptom(s)	OYES ONO OUnk
Loss of taste (Ageusia)	OYES ONO OUnk	If YES, specify:________________	
Seizures	OYES ONO OUnk		

VACCINATIONS

Covid-19 vaccination OYES ONO OUnk **Estimated date of most recent dose:**[_D_][_D_]/[_M_][_M_]/[_2_][_0_][_Y_][_Y_]

If YES, number of doses received: ________

If YES, specify type of the most recent vaccine: ________________________

If more than one dose has been given, specify all types of vaccine previously received:

____

Influenza vaccination within the last 6 months: OYES ONO OUnknown

PRE-ADMISSION MEDICATION *(taken within 14 days prior to admission/presentation at healthcare facility)*	
Steroids	OYES ONO OUnk If YES, OOral OInhaled OUnk
Other immunosuppressant agents (not oral steroids)	OYES ONO OUnk
Antibiotics	OYES ONO OUnk If YES, agent(s):________________
Antivirals	OYES ONO OUnk If YES, agent(s):________________
Other targeted COVID-19 Medications	OYES ONO OUnk If YES, agent(s):________________

SIGNS AND SYMPTOMS ON ADMISSION

Please provide details of clinical observations made as close to presentation/admission, or within 24 hours of admission. For observations not made immediately at admission, please record the first available data (patient reported and/or from medical records) within 24 hours of admission. For patients with a clear alternative diagnosis leading to admission who subsequently acquired COVID-19, complete these observations for the 24 hours after onset of symptoms of suspected or confirmed COVID-19 infection.

VACCINATIONS

If the exact date of the most recent dose of COVID-19 vaccine isn't available, please provide an estimate of the day the vaccine was given. Partial dates (e.g. Jan-2021) cannot be entered in the database.

PRE-ADMISSION MEDICATION *(taken within 14 days of admission/presentation at healthcare facility)*

Steroids: Examples include prednisolone, betamethasone, dexamethasone, hydrocortisone, methylprednisolone, deflazacort and fludrocortisone (oral), budesonide, fluticasone (inhaled).

Other immunosuppressant agents (not oral steroids): Examples include tofacitinib, cyclosporine, tacrolimus, sirolimus, everolimus, azathioprine, leflunomide, mycophenolate and biologics such as abatacept, adalimumab, anakinra, certolizumab, etanercept, adalimumab, infliximab and rituximab

Antibiotics: 'Antibiotic' refers to any agent(s) that selectively target bacteria. Please list generic names. Topical preparations should not be recorded.

Antivirals: Examples include ribavirin, lopinavir, ritonavir, remdesivir, oseltamivir, zanamivir, acyclovir, ganciclovir, and interferons. Please list generic names. Topical preparations should not be recorded.

Other targeted COVID-19 Medications: Includes for example: chloroquine, hydroxychloroquine, Interferon antibodies, convalescent plasma or any other COVID-19 therapeutics not included in the categories listed above. Please list generic names.

General Note: For free text entry of medications, please ensure correct spelling. For reference you may use: www.drugs.com

MODULE 1: PRESENTATION/ADMISSION CASE REPORT FORM

SIGNS AND SYMPTOMS AT HOSPITAL ADMISSION *(first available data at presentation/admission – within 24 hours)*

Temperature: [___][___][___].[___]O°C *or* O°F

HR: [___][___][___]beats/minute **RR:** [___][___]breaths/minute

Systolic BP: [___][___][___]mmHg **Diastolic BP:** [___][___][___]mmHg

Oxygen saturation: [___][___][___]% **On:** ORoom air OOxygen therapy OUnknown

Sternal capillary refill time >2sec. OYES ONO OUnknown **Height:** [___][___][___]cm **Weight:** [___][___][___]kg

SIGNS AND SYMPTOMS ON ADMISSION *(Unk = Unknown)*			
History of fever	OYES ONO OUnk	Fatigue / Malaise	OYES ONO OUnk
Cough OYES - non-productive	OYES - productive	Anorexia	OYES ONO OUnk
OYES - with haemoptysis	ONO OUnk	Altered consciousness/confusion	OYES ONO OUnk
Sore throat	OYES ONO OUnk	Muscle aches (myalgia)	OYES ONO OUnk
Runny nose (rhinorrhoea)	OYES ONO OUnk	Joint pain (arthralgia)	OYES ONO OUnk
Wheezing	OYES ONO OUnk	Inability to walk	OYES ONO OUnk
Shortness of breath	OYES ONO OUnk	Abdominal pain	OYES ONO OUnk
Lower chest wall indrawing	OYES ONO OUnk	Diarrhoea	OYES ONO OUnk
Chest pain	OYES ONO OUnk	Vomiting / Nausea	OYES ONO OUnk
Conjunctivitis	OYES ONO OUnk	Skin rash	OYES ONO OUnk
Lymphadenopathy	OYES ONO OUnk	Bleeding (Haemorrhage)	OYES ONO OUnk
Headache	OYES ONO OUnk	If YES, specify site(s):__________	
Loss of smell (Anosmia)	OYES ONO OUnk	Other symptom(s)	OYES ONO OUnk
Loss of taste (Ageusia)	OYES ONO OUnk	If YES, specify:__________	
Seizures	OYES ONO OUnk		

VACCINATIONS

Covid-19 vaccination OYES ONO OUnk **Estimated date of most recent dose:**[_D_][_D_]/[_M_][_M_]/[_2_][_0_][_Y_][_Y_]

If YES, number of doses received: ________

If YES, specify type of the most recent vaccine: __________

If more than one dose has been given, specify all types of vaccine previously received:

____

Influenza vaccination within the last 6 months: OYES ONO OUnknown

PRE-ADMISSION MEDICATION *(taken within 14 days prior to admission/presentation at healthcare facility)*	
Steroids	OYES ONO OUnk If YES, OOral OInhaled OUnk
Other immunosuppressant agents (not oral steroids)	OYES ONO OUnk
Antibiotics	OYES ONO OUnk If YES, agent(s):__________
Antivirals	OYES ONO OUnk If YES, agent(s):__________
Other targeted COVID-19 Medications	OYES ONO OUnk If YES, agent(s):__________

CO-MORBIDITIES AND RISK FACTORS

Please record if any of these comorbidities existed prior to admission.

In general, do not include past comorbidities that are no longer ongoing. Additional details are given below. Where example conditions are given, these are not intended to be exhaustive and other conditions of equivalent severity should be included.

Chronic cardiac disease (not hypertension)

Please include any of coronary artery disease, heart failure, congenital heart disease, cardiomyopathy, rheumatic heart disease.

Hypertension

Elevated arterial blood pressure diagnosed clinically, >140mmHg systolic or >90mmHg diastolic.

Chronic pulmonary disease (not asthma)

Please include any of chronic obstructive pulmonary disease (chronic bronchitis, chronic obstructive pulmonary disease (COPD), emphysema), cystic fibrosis, bronchiectasis, interstitial lung disease, pre-existing requirement for long term oxygen therapy. Do not include asthma.

Asthma (physician diagnosed)

Clinician-diagnosed asthma

Chronic Kidney Disease

Please include any of clinician-diagnosed chronic kidney disease, chronic estimated glomerular filtration rate < 60 mL/min/1.73m^2, history of kidney transplantation

Obesity (as defined by clinical staff)

This refers to patients for whom an attending clinician has assessed them to be obese - ideally but not necessarily with an objective measurement of obesity, such as calculation of the body mass index (BMI of 30 or more) or measurement of abdominal girth.

Moderate or severe liver disease

This is defined as cirrhosis with portal hypertension, with or without bleeding or a history of variceal bleeding.

Mild liver disease

This is defined as cirrhosis without portal hypertension or chronic hepatitis

Asplenia

Please include any of splenectomy, non-functional spleen, and congenital asplenia**.**

Chronic neurological disorder

Please include any of cerebral palsy, multiple sclerosis, motor neurone disease, muscular dystrophy, myasthenia gravis, Parkinson's disease, stroke, severe learning difficulty

Malignant neoplasm

Current solid organ or haematological malignancy. Please do not include malignancies that have been declared 'cured' ≥5 years ago with no evidence of ongoing disease. Do not include non-melanoma skin cancers. Do not include benign growths or dysplasia.

MODULE 1: PRESENTATION/ADMISSION CASE REPORT FORM

**CO-MORBIDITIES AND RISK FACTORS** *(existing prior to admission and ongoing)*			
Chronic cardiac disease *(not hypertension)*	○YES ○NO ○Unk	Chronic hematologic disease	○YES ○NO ○Unk
Hypertension	○YES ○NO ○Unk	AIDS / HIV ○YES-on ART ○YES-not on ART ○NO ○Unk<br>If YES, most recent CD4 count:<br>○< 200 ○200-< 500 ○≥ 500 cells/uL ○Unk	
Chronic pulmonary disease *(not asthma)*	○YES ○NO ○Unk	Diabetes Mellitus ○YES-Type 1 ○YES -Type 2<br>○YES -Gestational ○NO ○Unk<br>If YES, HbA1C results (within last 6 months) :________<br>Units: ○mmol/mol ○mmol/L ○%	
Asthma *(physician diagnosed)*	○YES ○NO ○Unk	Rheumatologic disorder	○YES ○NO ○Unk
Chronic kidney disease	○YES ○NO ○Unk	Dementia	○YES ○NO ○Unk
Obesity *(as defined by clinical staff)*	○YES ○NO ○Unk	Tuberculosis	○YES ○NO ○Unk
Moderate or severe liver disease	○YES ○NO ○Unk	Malnutrition	○YES ○NO ○Unk
Mild liver disease	○YES ○NO ○Unk	Smoking ○YES ○Never smoked ○Former smoker ○Unk	
Asplenia	○YES ○NO ○Unk	Other relevant risk factor(s)	○YES ○NO ○Unk
Chronic neurological disorder	○YES ○NO ○Unk	If YES, specify:	
Malignant neoplasm	○YES ○NO ○Unk		

MODULE 2: CASE REPORT FORM ON ADMISSION, CRITICAL CARE, RESEARCH SAMPLING

Complete on the day of admission or first COVID-19 investigation, and on the first day of ICU admission (if different from day of admission). In addition, complete for days when biochemical results are available.

SIGNS AND SYMPTOMS ***(Record the worst value between 00:00 to 24:00 on day of assessment)(worst=furthest from normal range)***

DATE OF ASSESSMENT *(DD/MM/YYYY)*: [_D_][_D_]/[_M_][_M_]/[_2_][_0_][_Y_][_Y_]

Highest temperature: [___][___][___].[___] ○°C *or* ○°F **HR:** [___][___][___]beats/minute **RR:** [___][___][___]breaths/minute

Systolic BP: [___][___][___]mmHg **Diastolic BP:** [___][___][___]mmHg

Oxygen saturation SaO_2 [___][___][___]%

Any supplemental oxygen: ○YES ○NO ○Unknown If yes,

FiO_2 ***(0.21-1.0)*** [___].[___][___] or [___][___] % or [___][___]L/min (Highest L/min)

PaO_2 *(at time nearest to the FiO_2 recorded at top of page)* [___][___][___]○kPa *or* ○mmHg ○Not done

PaO_2 sample type: ○Arterial ○Capillary ○Venous ○Unknown

From same blood gas record as PaO_2:

PCO_2 ___________○kPa *or* ○mmHg | **pH** ____________ | **HCO_3-** __________mEq/L | **Base excess** _________ mmol/L

Sternal capillary refill time >2seconds ○YES ○NO ○Unknown

AVPU: Alert [___] Verbal[___] Pain [___] Unresponsive [___] **Glasgow Coma Score (GCS / 15) [___][___]**

Richmond Agitation-Sedation Scale (RASS) [___]

Mean Arterial Blood Pressure [___][___][___]mmHg ○Unknown

Urine flow rate [___][___][___][___][___]mL/24 hours ○ Check if estimated ○Unknown

CO-MORBIDITIES, continued

Chronic hematologic disease

Any long-term disorder of the red or white blood cells, platelets or coagulation system requiring regular or intermittent treatment. Do not include leukaemia, lymphoma or myeloma, which should be entered under malignancy. Do not include iron-deficiency anaemia which is explained by diet or chronic blood loss.

AIDS/HIV

History of laboratory-confirmed HIV infection. Indicate whether or not the patient is on ART (antiretroviral therapy). Please provide the most recent CD4 count, if available.

Diabetes Mellitus

Type 1 or Type 2 diabetes mellitus requiring oral or subcutaneous treatment. Please indicate whether Type 1 or Type 2..If HbA1c results are available from the last 6 months only, please provide the most recent value.

Rheumatologic disorder

This is defined as an inflammatory and degenerative diseases of connective tissue structures. It includes chronic arthropathies and arthritis, connective tissue disorders and vasculitides.

Dementia

This is defined as clinical diagnosis of dementia

Tuberculosis

Patients currently receiving treatment for tuberculosis. Do not include latent tuberculosis.

Malnutrition

Any clinically identified deficiency in intake, either of total energy or of specific nutrients that led to a dietetic intervention or referral prior to the onset of COVID-19 symptoms. Do not include people who needed supplementary nutrition solely due to reduced intake during their current illness episode.

Smoking

Smoking at least one cigarette, cigar, pipe or equivalent per day before the onset of the current illness. Do not include smoke-free tobacco products such as chewed tobacco or electronic nicotine delivery devices.

Other relevant risk factor List any significant risk factors or comorbidities that existed prior to admission, are ongoing, that are not already listed.

MODULE 1: PRESENTATION/ADMISSION CASE REPORT FORM

**CO-MORBIDITIES AND RISK FACTORS** *(existing prior to admission and ongoing)*			
Chronic cardiac disease *(not hypertension)*	OYES ONO OUnk	Chronic hematologic disease	OYES ONO OUnk
Hypertension	OYES ONO OUnk	AIDS / HIV OYES-on ART OYES-not on ART ONO OUnk<br>If YES, most recent CD4 count:<br>O< 200 O200-< 500 O≥ 500 cells/uL OUnk	
Chronic pulmonary disease *(not asthma)*	OYES ONO OUnk	Diabetes Mellitus OYES-Type 1 OYES -Type 2 OYES -Gestational ONO OUnk<br>If YES, HbA1C results (within last 6 months) :________<br>Units: Ommol/mol Ommol/L O%	
Asthma *(physician diagnosed)*	OYES ONO OUnk	Rheumatologic disorder	OYES ONO OUnk
Chronic kidney disease	OYES ONO OUnk	Dementia	OYES ONO OUnk
Obesity *(as defined by clinical staff)*	OYES ONO OUnk	Tuberculosis	OYES ONO OUnk
Moderate or severe liver disease	OYES ONO OUnk	Malnutrition	OYES ONO OUnk
Mild liver disease	OYES ONO OUnk	Smoking OYES ONever smoked OFormer smoker OUnk	
Asplenia	OYES ONO OUnk	Other relevant risk factor(s)	OYES ONO OUnk
Chronic neurological disorder	OYES ONO OUnk	If YES, specify:	
Malignant neoplasm	OYES ONO OUnk		

MODULE 2: CASE REPORT FORM ON ADMISSION, CRITICAL CARE, RESEARCH SAMPLING

Complete on the day of admission or first COVID-19 investigation, and on the first day of ICU admission (if different from day of admission). In addition, complete for days when biochemical results are available.

SIGNS AND SYMPTOMS ***(Record the worst value between 00:00 to 24:00 on day of assessment)(worst=furthest from normal range)***

DATE OF ASSESSMENT *(DD/MM/YYYY)*: [_D_][_D_]/[_M_][_M_]/[_2_][_0_][_Y_][_Y_]

Highest temperature: [___][___][___].[___] O°C *or* O°F **HR:** [___][___][___]beats/minute **RR:** [___][___][___]breaths/minute

Systolic BP: [___][___][___]mmHg **Diastolic BP:** [___][___][___]mmHg

Oxygen saturation SaO_2 [___][___][___]%

Any supplemental oxygen: OYES ONO OUnknown If yes,

FiO_2 ***(0.21-1.0)*** [___].[___][___] or [___][___] % or [___][___]L/min (Highest L/min)

PaO_2 *(at time nearest to the FiO_2 recorded at top of page)* [___][___][___]OkPa *or* OmmHg ONot done

PaO_2 sample type: OArterial OCapillary OVenous OUnknown

From same blood gas record as PaO_2:

PCO_2 ____________OkPa *or* OmmHg | **pH** _____________ | **HCO_3-** ___________mEq/L | **Base excess** __________ mmol/L

Sternal capillary refill time >2seconds OYES ONO OUnknown

AVPU: Alert [___] **Verbal**[___] **Pain** [___] **Unresponsive** [___] **Glasgow Coma Score (GCS / 15)** [___][___]

Richmond Agitation-Sedation Scale (RASS) [___]

Mean Arterial Blood Pressure [___][___][___]mmHg OUnknown

Urine flow rate [___][___][___][___][___]mL/24 hours O Check if estimated OUnknown

MODULE 2 CASE REPORT FORM ON ADMISSION, CRITICAL CARE, RESEARCH SAMPLING

SIGNS AND SYMPTOMS

Highest Temperature

Please enter the highest peripheral body temperature (rectal if < 3 months) recorded during the course of the day in the space provided and indicate the unit of measurement, either degrees Celsius (°C) or Fahrenheit (°F).

Heart rate (HR)

Enter the heart rate measured in beats per minute. This may be measured manually or by electronic monitoring.

Respiratory rate (RR)

Enter the respiratory rate in breaths per minute. Manual rather than electronic measurement is preferred where possible (this is achieved by counting the number of breaths for one minute, counting how many times the chest rises within this time period). If both abnormal low and high rate observed, record the abnormally high rate.

Systolic BP

Please report the systolic and diastolic blood pressure from the observation with the lowest mean arterial pressure (if mean arterial pressure has not been calculated, report the measurement with lowest systolic blood pressure).

Please enter the systolic blood pressure measured in millimetres of mercury (mmHg), in the relevant sections. For example, if the blood pressure is 120/85 mmHg, enter 120 in the section marked 'systolic BP'. Use any recognised method for measuring blood pressure.

Diastolic BP

Please enter the diastolic blood pressure measured in millimetres of mercury (mmHg), in the relevant sections. For example, if the blood pressure is 120/85 mmHg, enter 85 in the section marked 'diastolic BP'. Use any recognised method for measuring blood pressure.

Oxygen saturation SaO_2

For all patients, irrespective of ventilation or supplemental oxygen requirement, please enter the percentage oxygen saturation. This may be measured by pulse oximetry or by arterial blood gas analysis.

Any supplemental oxygen: FiO_2 (0.21-1.0)

This is a key indicator to complete for all patients. If the patient received any form of supplemental oxygen through a mask or nasal cannula that delivers a known concentration of oxygen or is being ventilated, please provide the fraction of inspired oxygen (FiO_2) delivered. For patients receiving oxygen through any means, such as a face mask or nasal cannula, that does not deliver a known oxygen concentration provide the maximum flow rate received on day of completion in L/min.

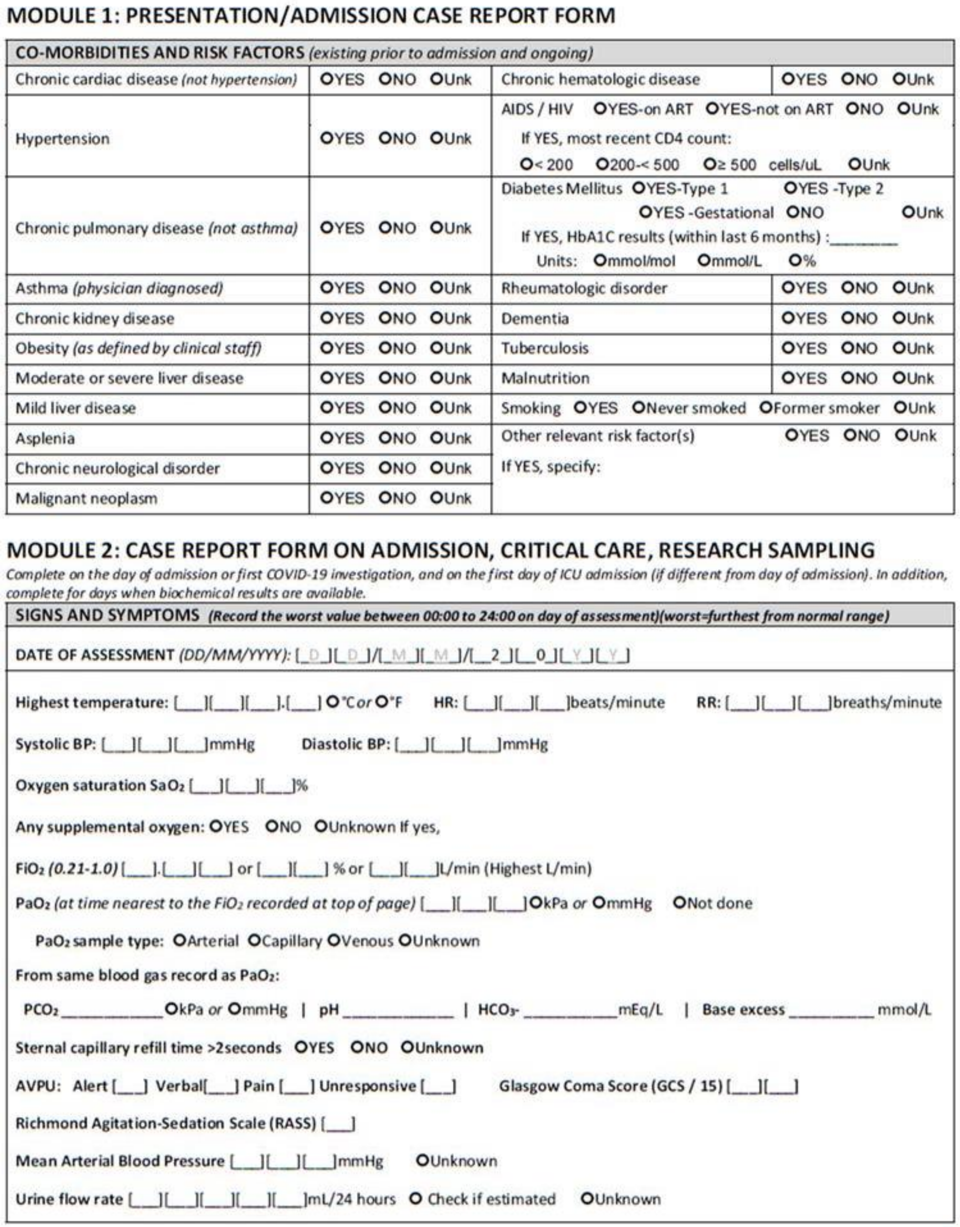

MODULE 1: PRESENTATION/ADMISSION CASE REPORT FORM

CO-MORBIDITIES AND RISK FACTORS *(existing prior to admission and ongoing)*			
Chronic cardiac disease *(not hypertension)*	○YES ○NO ○Unk	Chronic hematologic disease	○YES ○NO ○Unk
Hypertension	○YES ○NO ○Unk	AIDS / HIV ○YES-on ART ○YES-not on ART ○NO ○Unk<br>If YES, most recent CD4 count:<br>○< 200 ○200-< 500 ○≥ 500 cells/uL ○Unk	
Chronic pulmonary disease *(not asthma)*	○YES ○NO ○Unk	Diabetes Mellitus ○YES-Type 1 ○YES -Type 2<br>○YES -Gestational ○NO ○Unk<br>If YES, HbA1C results (within last 6 months) :________<br>Units: ○mmol/mol ○mmol/L ○%	
Asthma *(physician diagnosed)*	○YES ○NO ○Unk	Rheumatologic disorder	○YES ○NO ○Unk
Chronic kidney disease	○YES ○NO ○Unk	Dementia	○YES ○NO ○Unk
Obesity *(as defined by clinical staff)*	○YES ○NO ○Unk	Tuberculosis	○YES ○NO ○Unk
Moderate or severe liver disease	○YES ○NO ○Unk	Malnutrition	○YES ○NO ○Unk
Mild liver disease	○YES ○NO ○Unk	Smoking ○YES ○Never smoked ○Former smoker ○Unk	
Asplenia	○YES ○NO ○Unk	Other relevant risk factor(s) ○YES ○NO ○Unk<br>If YES, specify:	
Chronic neurological disorder	○YES ○NO ○Unk		
Malignant neoplasm	○YES ○NO ○Unk		

MODULE 2: CASE REPORT FORM ON ADMISSION, CRITICAL CARE, RESEARCH SAMPLING

Complete on the day of admission or first COVID-19 investigation, and on the first day of ICU admission (if different from day of admission). In addition, complete for days when biochemical results are available.

SIGNS AND SYMPTOMS ***(Record the worst value between 00:00 to 24:00 on day of assessment)(worst=furthest from normal range)***

DATE OF ASSESSMENT *(DD/MM/YYYY)*: [_D_][_D_]/[_M_][_M_]/[_2_][_0_][_Y_][_Y_]

Highest temperature: [___][___][___].[___] ○°C *or* ○°F **HR:** [___][___][___]beats/minute **RR:** [___][___][___]breaths/minute

Systolic BP: [___][___][___]mmHg **Diastolic BP:** [___][___][___]mmHg

Oxygen saturation SaO_2 [___][___][___]%

Any supplemental oxygen: ○YES ○NO ○Unknown If yes,

FiO_2 *(0.21-1.0)* [___].[___][___] or [___][___] % or [___][___]L/min (Highest L/min)

PaO_2 *(at time nearest to the FiO_2 recorded at top of page)* [___][___][___]○kPa *or* ○mmHg ○Not done

PaO_2 sample type: ○Arterial ○Capillary ○Venous ○Unknown

From same blood gas record as PaO_2:

PCO_2 ___________○kPa *or* ○mmHg | pH ____________ | HCO_3- __________mEq/L | Base excess _________ mmol/L

Sternal capillary refill time >2seconds ○YES ○NO ○Unknown

AVPU: Alert [___] Verbal[___] Pain [___] Unresponsive [___] Glasgow Coma Score (GCS / 15) [___][___]

Richmond Agitation-Sedation Scale (RASS) [___]

Mean Arterial Blood Pressure [___][___][___]mmHg ○Unknown

Urine flow rate [___][___][___][___][___]mL/24 hours ○ Check if estimated ○Unknown

SIGNS AND SYMPTOMS, continued

PaO_2 (*at time nearest to the FiO_2 recorded at top of page*)

PaO_2 (partial pressure of oxygen in blood) as determined by arterial/ capillary blood gas analysis. This PaO_2 must correspond with the oxygen therapy documented in the FiO_2 field. Please fill in the lowest value in either mmHg or kPa depending on the output of your blood gas analyser. If the PaO_2 is not known, place NA in the data field.

From the same blood gas record as PaO_2:

$PaCO_2$ is the partial pressure of carbon dioxide measured in the sample. pH is the measure of the activity of the (solvated) hydrogen ion (H+) measured in the sample. HCO_3- refers to the bicarbonate measured in the blood gas sample. Base excess refers to standardised base excess (SBE). If standardised base excess is not reported, enter the base excess value presented, this can be either a positive or negative value.

Sternal capillary refill time > 2 seconds?

Sternal capillary refill time is measured by pressing on the sternum for five seconds with a finger or thumb until the underlying skin turns white and then noting the time in seconds needed for the colour to return once the pressure is released.

AVPU

Alert – responding to voice – responding to pain – unresponsive: please state the least responsive condition of the patient during the calendar day (not counting normal sleep). On day of admission record the value as close to admission as possible before treatments have been administered. For daily records, if the patient is being sedated on the day of assessment record the value before the sedation.

Glasgow Coma Score (GCS / 15)

Please state the lowest GCS recorded. For intubated patients and patients with a non-fenestrated tracheostomy, give 1 point for the voice component and calculate the total as usual. Suffixes such as t for tracheostomy cannot be entered on to the database. If the patient is sedated on the day of assessment these parameters should correspond to the values observed before sedation. For daily recording, if the patient is fully sedated for the duration of the day of assessment (from 00:00 to 24:00) record non testable. Glasgow Coma Score: https://www.glasgowcomascale.org/downloads/GCS-Assessment-Aid-English.pdf?v=3

Richmond Agitation-Sedation Scale (RASS)

RASS – If done, enter the lowest calculated value (between -5 and 4) on the date of assessment.

MODULE 1: PRESENTATION/ADMISSION CASE REPORT FORM

CO-MORBIDITIES AND RISK FACTORS *(existing prior to admission and ongoing)*			
Chronic cardiac disease *(not hypertension)*	OYES ONO OUnk	Chronic hematologic disease	OYES ONO OUnk
Hypertension	OYES ONO OUnk	AIDS / HIV OYES-on ART OYES-not on ART ONO OUnk<br>If YES, most recent CD4 count:<br>O< 200 O200-< 500 O≥ 500 cells/uL OUnk	
Chronic pulmonary disease *(not asthma)*	OYES ONO OUnk	Diabetes Mellitus OYES-Type 1 OYES -Type 2 OYES -Gestational ONO OUnk<br>If YES, HbA1C results (within last 6 months) :________<br>Units: Ommol/mol Ommol/L O%	
Asthma *(physician diagnosed)*	OYES ONO OUnk	Rheumatologic disorder	OYES ONO OUnk
Chronic kidney disease	OYES ONO OUnk	Dementia	OYES ONO OUnk
Obesity *(as defined by clinical staff)*	OYES ONO OUnk	Tuberculosis	OYES ONO OUnk
Moderate or severe liver disease	OYES ONO OUnk	Malnutrition	OYES ONO OUnk
Mild liver disease	OYES ONO OUnk	Smoking OYES ONever smoked OFormer smoker OUnk	
Asplenia	OYES ONO OUnk	Other relevant risk factor(s)	OYES ONO OUnk
Chronic neurological disorder	OYES ONO OUnk	If YES, specify:	
Malignant neoplasm	OYES ONO OUnk		

MODULE 2: CASE REPORT FORM ON ADMISSION, CRITICAL CARE, RESEARCH SAMPLING

Complete on the day of admission or first COVID-19 investigation, and on the first day of ICU admission (if different from day of admission). In addition, complete for days when biochemical results are available.

SIGNS AND SYMPTOMS ***(Record the worst value between 00:00 to 24:00 on day of assessment)(worst=furthest from normal range)***

DATE OF ASSESSMENT *(DD/MM/YYYY)*: [_D_][_D_]/[_M_][_M_]/[_2_][_0_][_Y_][_Y_]

Highest temperature: [___][___][___].[___] O°C or O°F **HR:** [___][___][___]beats/minute **RR:** [___][___][___]breaths/minute

Systolic BP: [___][___][___]mmHg **Diastolic BP:** [___][___][___]mmHg

Oxygen saturation SaO_2 [___][___][___]%

Any supplemental oxygen: OYES ONO OUnknown If yes,

FiO_2 *(0.21-1.0)* [___].[___][___] or [___][___] % or [___][___]L/min (Highest L/min)

PaO_2 *(at time nearest to the FiO_2 recorded at top of page)* [___][___][___]OkPa *or* OmmHg ONot done

PaO_2 sample type: OArterial OCapillary OVenous OUnknown

From same blood gas record as PaO_2:

PCO_2 __________OkPa *or* OmmHg | **pH** __________ | **HCO_3-** __________mEq/L | **Base excess** __________ mmol/L

Sternal capillary refill time >2seconds OYES ONO OUnknown

AVPU: Alert [___] Verbal[___] Pain [___] Unresponsive [___] **Glasgow Coma Score (GCS / 15)** [___][___]

Richmond Agitation-Sedation Scale (RASS) [___]

Mean Arterial Blood Pressure [___][___][___]mmHg OUnknown

Urine flow rate [___][___][___][___][___]mL/24 hours O Check if estimated OUnknown

Current admission to ICU/ITU/IMC/HDU?

If the patient has been admitted to an intensive care, intensive therapy, intermediate care or high dependency unit please tick ‘yes’. If the patient is on a general care ward then select ‘no’ or ‘Unknown’.

See Outcome Case Report Form (below) for guidelines on recording treatment data

LABORATORY RESULTS

Please record all laboratory results available on day of admission, or the day that COVID-19 was first clinically suspected in patients already admitted to hospital, and on day of admission to ICU/HDU.
For daily records: record the date of assessment as the day the blood sample/s were taken.. If the unit of measurement is not shown on the paper form it will likely appear in the dropdown list in the eCRF. If you cannot find the correct unit on the eCRF please use a unit converter, such as: http://unitslab.com/ or equivalent or email ncov@isaric.org to let us know.
‘Worst value’ refers to values furthest from the normal physiological range or laboratory normal range. Results that were rejected by the clinical team (e.g. haemolysed blood samples, contaminated microbiology results) should not be reported.

Haemoglobin (Hb or Hgb) refers to haemoglobin concentration measurement in blood.

WBC count is the total white blood cell count in blood.

Haematocrit (Ht or HCT), also known as packed cell volume (PCV) or erythrocyte volume fraction (EVF), is the volume percentage (%) of red blood cells in blood.

APTT is the activated partial thromboplastin time. Record the highest value.

APTR is the activated partial thromboplastin ratio. Record the highest value.

PT is the prothrombin time. Record the highest value.

INR is the international normalised ratio. Record the highest value.

ALT/SGPT: ALT is alanine transaminase (also called serum glutamic pyruvate transaminase, SGPT). Record the highest value.

Total Bilirubin refers to total bilirubin measured in the blood. Record the highest value.

AST/SGOT is aspartate transaminase (also called serum glutamic oxaloacetic transaminase, SGOT). Record the highest value.

Glucose refers to blood glucose test. Random glucose measurement is preferred to a fasted measurement.

Blood urea nitrogen is also known as ‘urea’, measured in a blood sample. Record the highest value.

Lactate refers to blood lactate. Record the highest value.

Creatinine refers to serum creatinine. Record the highest value.

Procalcitonin or PCT refers to blood procalcitonin. Record the highest value.

CRP is C-reactive protein and refers to the blood (serum or plasma) CRP level. Record the highest value.

MODULE 2: CASE REPORT FORM ON ADMISSION, CRITICAL CARE, RESEARCH SAMPLING

Complete on the day of admission or first COVID-19 investigation, and on the first day of ICU admission (if different from day of admission). In addition, depending on available resources, complete every day for a maximum of 14 days, or for days when biochemical results are available.

Is the patient currently receiving, or has received *(between 00:00 to 24:00 on day of assessment)*

Current admission to ICU/ITU/IMC/HDU? ○YES ○NO ○Unknown

High-flow nasal cannula oxygen therapy? ○YES ○NO ○Unknown

Non-invasive ventilation *(Any)*? ○YES ○NO ○Unknown If YES: ○BIPAP ○CPAP ○Other ○Unknown

Invasive ventilation? ○YES ○NO ○Unknown

Prone positioning? ○YES ○NO ○Unknown If yes, ○during invasive ventilation ○whilst self-ventilating ○Unknown

Inhaled Nitric Oxide? ○YES ○NO ○Unknown

Tracheostomy inserted? ○YES ○NO ○Unknown

Extra corporeal life support (ECLS/ ECMO)? ○YES ○NO ○Unknown If YES: ○VV ○AV ○Central ○Unknown

Renal replacement therapy (RRT) or dialysis? ○YES ○NO ○Unknown

Any vasopressor/inotropic support? ○YES ○NO ○Unknown *(if NO, select NO for the next 3 questions)*

Dopamine <5µg/kg/min OR Dobutamine OR milrinone OR levosimendan: ○YES ○NO

Dopamine 5-15µg/kg/min OR Epinephrine/Norepinephrine < 0.1µg/kg/min OR vasopressin OR phenylephrine: ○YES ○NO

Dopamine >15µg/k/min OR Epinephrine/Norepinephrine > 0.1µg/kg/min: ○YES ○NO

Neuromuscular blocking agents? ○YES ○NO ○Unknown

Other intervention(s) or procedure(s)? ○YES ○NO ○Unknown If YES, Specify: ______________________

____

LABORATORY RESULTS (on admission, on any admission to ICU, then daily) – complete every line

DATE OF ASSESSMENT *(DD/MM/YYYY)*: [_D_][_D_]/[_M_][_M_]/[_2_][_0_][_Y_][_Y_]

LABORATORY RESULTS (**record units if different from those listed)*
Record the worst value between 00:00 to 24:00 on day of assessment (if Not Available write ‘N/A’)

Parameter	Value*	Not done	Parameter	Value*	Not done
Haemoglobin (g/L)		○	Urea (BUN) (mmol/L)		○
WBC count (x10^9/L)		○	Lactate (mmol/L)		○
Lymphocyte count (10^9/L)		○	Creatinine (µmol/L)		○
Neutrophil count (10^9/L)		○	Sodium (mmol/L)		○
Haematocrit (%)		○	Potassium (mmol/L)		○
Platelets (x10^9/L)		○	Procalcitonin (ng/mL)		○
APTT (seconds))		○	CRP (mg/L)		○
APTR		○	LDH (U/L)		○
PT (seconds)		○	Creatine kinase (U/L)		○
INR		○	Troponin I (ng/mL)		○
ALT/SGPT (U/L)		○	D-dimer (mg/L)		○
Total bilirubin (µmol/L)		○	Ferritin (ng/mL)		○
AST/SGOT (U/L)		○	IL-6 (pg/mL)		○
Glucose (mmol/L)		○	Fibrinogen (mg/dl)		○

LDH is lactate dehydrogenase. Record the highest value.

Creatine kinase (CK, or creatine phosphokinase, CPK) refers to total creatine kinase measured in the blood. Record the highest value.

Troponin I Record the highest value

D-dimer Record the highest value

Ferritin Record the highest value

IL-6 is Interleukin 6. Record the highest value

MODULE 2: CASE REPORT FORM ON ADMISSION, CRITICAL CARE, RESEARCH SAMPLING

Complete on the day of admission or first COVID-19 investigation, and on the first day of ICU admission (if different from day of admission). In addition, depending on available resources, complete every day for a maximum of 14 days, or for days when biochemical results are available.

Is the patient currently receiving, or has received *(between 00:00 to 24:00 on day of assessment)*

Current admission to ICU/ITU/IMC/HDU? OYES ONO OUnknown

High-flow nasal cannula oxygen therapy? OYES ONO OUnknown

Non-invasive ventilation *(Any)*? OYES ONO OUnknown If YES: OBIPAP OCPAP OOther OUnknown

Invasive ventilation? OYES ONO OUnknown

Prone positioning? OYES ONO OUnknown If yes, Oduring invasive ventilation Owhilst self-ventilating OUnknown

Inhaled Nitric Oxide? OYES ONO OUnknown

Tracheostomy inserted? OYES ONO OUnknown

Extra corporeal life support (ECLS/ ECMO)? OYES ONO OUnknown If YES: OVV OAV OCentral OUnknown

Renal replacement therapy (RRT) or dialysis? OYES ONO OUnknown

Any vasopressor/inotropic support? OYES ONO OUnknown *(if NO, select NO for the next 3 questions)*

Dopamine <5µg/kg/min OR Dobutamine OR milrinone OR levosimendan: OYES ONO

Dopamine 5-15µg/kg/min OR Epinephrine/Norepinephrine < 0.1µg/kg/min OR vasopressin OR phenylephrine: OYES ONO

Dopamine >15µg/k/min OR Epinephrine/Norepinephrine > 0.1µg/kg/min: OYES ONO

Neuromuscular blocking agents? OYES ONO OUnknown

Other intervention(s) or procedure(s)? OYES ONO OUnknown If YES, Specify: ____________________

LABORATORY RESULTS (on admission, on any admission to ICU, then daily) – complete every line

DATE OF ASSESSMENT *(DD/MM/YYYY)*: [_D_][_D_]/[_M_][_M_]/[_2_][_0_][_Y_][_Y_]

LABORATORY RESULTS (**record units if different from those listed)*

Record the worst value between 00:00 to 24:00 on day of assessment (if Not Available write 'N/A')

Parameter	Value*	Not done	Parameter	Value*	Not done
Haemoglobin (g/L)		O	Urea (BUN) (mmol/L)		O
WBC count (x10^9/L)		O	Lactate (mmol/L)		O
Lymphocyte count (10^9/L)		O	Creatinine (µmol/L)		O
Neutrophil count (10^9/L)		O	Sodium (mmol/L)		O
Haematocrit (%)		O	Potassium (mmol/L)		O
Platelets (x10^9/L)		O	Procalcitonin (ng/mL)		O
APTT (seconds))		O	CRP (mg/L)		O
APTR		O	LDH (U/L)		O
PT (seconds)		O	Creatine kinase (U/L)		O
INR		O	Troponin I (ng/mL)		O
ALT/SGPT (U/L)		O	D-dimer (mg/L)		O
Total bilirubin (µmol/L)		O	Ferritin (ng/mL)		O
AST/SGOT (U/L)		O	IL-6 (pg/mL)		O
Glucose (mmol/L)		O	Fibrinogen (mg/dl)		O

MODULE 3: OUTCOME CASE REPORT FORM

TREATMENT

For all questions of duration, please count the number of calendar days that the patient received the treatment. For treatments that were stopped and restarted, count those days on which the treatment was given but don't count any calendar days on which it was not given at all.

Oxygen therapy

Complete this field for all patients. If the patient received any form of supplementary oxygen, via nose cannula, mask or non-invasive or invasive ventilation tick 'yes' and indicate the total days they received any form of oxygen (O_2) therapy.

If any supplemental oxygen (at any concentration) was given by any means of delivery **at any point** during the patient's hospital stay, place a cross in the box marked 'yes'. This includes any supplementary oxygen (O_2) delivered via non-invasive facemasks/nasal cannula/mask or via invasive mechanical ventilation. Please also indicate the maximum O_2 flow volume. If it is not possible to access record of the absolute highest O_2 volume delivered during the admission indicate the highest known.

Non-invasive ventilation (Any)

If the patient received non-invasive ventilation (NIV), defined as the provision of ventilatory support through the patient's upper airway using a mask or similar device, at any time during their hospital stay, place tick 'yes' and enter the total duration in days if known.

Invasive ventilation (Any)

Invasive ventilation means that patient has undergone tracheal intubation, for the purpose of invasive mechanical ventilation. Invasive ventilation is a method to mechanically assist or replace spontaneous breathing in patients by use of a powered device that forces oxygenated air into the lungs. The mode of intubation may be orotracheal, nasotracheal, or via a cricothyrotomy or tracheotomy.

Prone Positioning

Prone ventilation refers to ventilation with the patient lying in the prone position. If the patient received prone ventilation at any time during their hospital stay, please tick 'yes' and indicate the total duration in days.

Renal replacement therapy (RRT) or dialysis

Renal replacement therapy includes haemodialysis, peritoneal dialysis (PD), intermittent haemodialysis (IHD), on-line intermittent haemofiltration (IHF), on-line haemodiafiltration (IHDF), continuous haemofiltration (CHF) and continuous haemodiafiltration (CHDF), continuous venovenous haemofiltration (CVVH), continuous venovenous haemodialysis (CVVHD), continuous venovenous haemodiafiltration (CVVHDF), slow continuous ultrafiltration (SCUF), continuous arteriovenous haemofiltration (CAVHD), sustained low-efficiency dialysis (SLED) and continuous renal replacement therapy (CRRT)

Inotropes/vasopressors?

A vasopressor is a pharmaceutical agent that causes vasoconstriction. Agents include norepinephrine, epinephrine, vasopressin, terlipressin and phenylephrine. An inotrope is a pharmaceutical agent that alters the force of myocardial contractility. Commonly used 'positive' inotropes include dobutamine, dopamine, milrinone and adrenaline (epinephrine). If the patient received a vasopressor or inotrope for at least one hour during their hospital stay, place tick 'yes' and the total duration in days if known.

MODULE 3: OUTCOME CASE REPORT FORM

TREATMENT: At ANY time during hospitalisation, did the patient receive/undergo:

Any Oxygen therapy? O YES O NO O Unknown   If YES, total duration: ________days O Unknown

Maximum O_2 flow volume: O <2 L/min O 2-5 L/min O 6-10 L/min O 11-15 L/min O >15 L/min

Non-invasive ventilation? *(Any)* O YES O NO O Unknown   If YES, total duration: ________days O Unknown

Invasive ventilation? *(Any)* O YES O NO O Unknown   If YES, total duration: ________days O Unknown

High flow nasal oxygen O YES O NO O Unknown   If YES, total duration: ________days O Unknown

Prone Positioning? O YES O NO O Unknown

Inhaled Nitric Oxide? O YES O NO O Unknown

Tracheostomy inserted? O YES O NO O Unknown

Extracorporeal support (ECMO)? O YES O NO O Unknown   If YES, total duration: ________days O Unknown

Renal replacement therapy (RRT) or dialysis? O YES O NO O Unknown

Inotropes/vasopressors? O YES O NO O Unknown

ICU or High Dependency Unit admission? O YES O NO O Unknown   If YES, total duration: ________days O Unknown

If YES, date of ICU admission: [_D_][_D_]/[_M_][_M_]/[_2_][_0_][_Y_][_Y_] O Unknown

date of ICU discharge: [_D_][_D_]/[_M_][_M_]/[_2_][_0_][_Y_][_Y_] O Unknown

COMPLICATIONS: At any time during hospitalisation did the patient experience: *(Unk = Unknown)*

Viral pneumonia/pneumonitis	O YES O NO O Unk	Meningitis / Encephalitis	O YES O NO O Unk
Bacterial pneumonia	O YES O NO O Unk	Bacteremia	O YES O NO O Unk
Acute Respiratory Distress Syndrome	O YES O NO O Unk	Coagulation disorder / DIC	O YES O NO O Unk
Pneumothorax	O YES O NO O Unk	Pulmonary Embolism	O YES O NO O Unk
Pleural effusion	O YES O NO O Unk	Deep Vein Thrombosis	O YES O NO O Unk
Cryptogenic organizing pneumonia (COP)	O YES O NO O Unk	Other thromboembolism (not PE or DVT)	O YES O NO O Unk
Bronchiolitis	O YES O NO O Unk	Anemia	O YES O NO O Unk
Cardiac arrest	O YES O NO O Unk	Rhabdomyolysis / Myositis	O YES O NO O Unk
Myocardial infarction	O YES O NO O Unk	Acute renal injury/ Acute renal failure	O YES O NO O Unk
Cardiac ischaemia	O YES O NO O Unk	Gastrointestinal haemorrhage	O YES O NO O Unk
Cardiac arrhythmia	O YES O NO O Unk	Pancreatitis	O YES O NO O Unk
Myocarditis / Pericarditis	O YES O NO O Unk	Liver dysfunction	O YES O NO O Unk
Endocarditis	O YES O NO O Unk	Hyperglycemia	O YES O NO O Unk
Cardiomyopathy	O YES O NO O Unk	Hypoglycemia	O YES O NO O Unk
Congestive heart failure	O YES O NO O Unk	Other	O YES O NO O Unk
Seizure	O YES O NO O Unk	If YES, specify:______________________	
Stroke / Cerebrovascular accident	O YES O NO O Unk		

COMPLICATIONS

Please select all that were clinically identified at any time during the hospital admission.
Do not include known comorbidities (e.g. previous atrial fibrillation should not be included but new onset during this admission should). Record physician diagnosed complications.

Viral pneumonitis/pneumonia
Clinically or radiologically diagnosed viral pneumonitis/pneumonia.

Bacterial pneumonia
Clinically or radiologically diagnosed bacterial pneumonia (including community, hospital and ventilator acquired) managed with antimicrobials. Bacteriological confirmation not required.

Acute Respiratory Distress Syndrome (ARDS)
Defined according to Berlin criteria as:

- Occurring within 1 week of a known clinical insult or worsening respiratory symptoms
- Bilateral radiological opacities not fully explained by effusions, lobar/lung collapse, or nodules
- Respiratory failure not fully explained by cardiac failure or fluid overload

Pneumothorax
Is defined as the abnormal presence of air in the pleural cavity (between the lungs and the chest wall), causing collapse of the lung. It may be diagnosed clinically, usually with radiological confirmation.

Pleural effusion
Is defined as increased amounts of fluid within the pleural cavity. It may be diagnosed clinically, with or without radiological or interventional confirmation.

Cryptogenic organizing pneumonia (COP)
Idiopathic diffuse interstitial lung disease, diagnosed radiologically (multiple consolidative or ground glass opacities) or histologically (granulation tissue and chronic inflammatory infiltrate in alveoli). Formerly known as bronchiolitis obliterans organizing pneumonia (BOOP)

Bronchiolitis
This is a clinical diagnosis.

Cardiac arrest
Sudden cessation of cardiac activity with no normal breathing and no signs of circulation.

Myocardial infarction
Myocardial ischaemia (MI) leading to injury/necrosis, diagnosed by clinical findings, altered electrocardiography and elevated cardiac enzymes.

Cardiac ischaemia
Is defined as diminished blood and oxygen supply to the heart muscle, also known as myocardial ischemia, It is confirmed by an electrocardiogram (showing ischaemic changes, e.g. ST depression or elevation) and/or cardiac enzyme elevation.

Cardiac arrhythmia
If a cardiac arrhythmia is identified and there is no previous record of it, select 'yes'.

MODULE 3: OUTCOME CASE REPORT FORM

TREATMENT: At ANY time during hospitalisation, did the patient receive/undergo:

Any Oxygen therapy? OYES ONO OUnknown If YES, total duration: _________days OUnknown

Maximum O₂ flow volume: O <2 L/min O2-5 L/min O6-10 L/min O11-15 L/min O>15 L/min

Non-invasive ventilation? *(Any)* OYES ONO OUnknown If YES, total duration: _________days OUnknown

Invasive ventilation? *(Any)* OYES ONO OUnknown If YES, total duration: _________days OUnknown

High flow nasal oxygen OYES ONO OUnknown If YES, total duration: _________days OUnknown

Prone Positioning? OYES ONO OUnknown

Inhaled Nitric Oxide? OYES ONO OUnknown

Tracheostomy inserted? OYES ONO OUnknown

Extracorporeal support (ECMO)? OYES ONO OUnknown If YES, total duration: _________days OUnknown

Renal replacement therapy (RRT) or dialysis? OYES ONO OUnknown

Inotropes/vasopressors? OYES ONO OUnknown

ICU or High Dependency Unit admission? OYES ONO OUnknown If YES, total duration: _________days OUnknown

If YES, date of ICU admission: [_D_][_D_]/[_M_][_M_]/[_2_][_0_][_Y_][_Y_] OUnknown

date of ICU discharge: [_D_][_D_]/[_M_][_M_]/[_2_][_0_][_Y_][_Y_] OUnknown

COMPLICATIONS: At any time during hospitalisation did the patient experience: *(Unk = Unknown)*

Viral pneumonia/pneumonitis	OYES ONO OUnk	Meningitis / Encephalitis	OYES ONO OUnk
Bacterial pneumonia	OYES ONO OUnk	Bacteremia	OYES ONO OUnk
Acute Respiratory Distress Syndrome	OYES ONO OUnk	Coagulation disorder / DIC	OYES ONO OUnk
Pneumothorax	OYES ONO OUnk	Pulmonary Embolism	OYES ONO OUnk
Pleural effusion	OYES ONO OUnk	Deep Vein Thrombosis	OYES ONO OUnk
Cryptogenic organizing pneumonia (COP)	OYES ONO OUnk	Other thromboembolism (not PE or DVT)	OYES ONO OUnk
Bronchiolitis	OYES ONO OUnk	Anemia	OYES ONO OUnk
Cardiac arrest	OYES ONO OUnk	Rhabdomyolysis / Myositis	OYES ONO OUnk
Myocardial infarction	OYES ONO OUnk	Acute renal injury/ Acute renal failure	OYES ONO OUnk
Cardiac ischaemia	OYES ONO OUnk	Gastrointestinal haemorrhage	OYES ONO OUnk
Cardiac arrhythmia	OYES ONO OUnk	Pancreatitis	OYES ONO OUnk
Myocarditis / Pericarditis	OYES ONO OUnk	Liver dysfunction	OYES ONO OUnk
Endocarditis	OYES ONO OUnk	Hyperglycemia	OYES ONO OUnk
Cardiomyopathy	OYES ONO OUnk	Hypoglycemia	OYES ONO OUnk
Congestive heart failure	OYES ONO OUnk	Other	OYES ONO OUnk
Seizure	OYES ONO OUnk	If YES, specify:______________________	
Stroke / Cerebrovascular accident	OYES ONO OUnk		

COMPLICATIONS, continued

Myocarditis / Pericarditis

Myocarditis / pericarditis refers to an inflammation of the heart or pericardium (outer lining of the heart). Diagnosis can be clinical, biochemical (cardiac enzymes) or radiological

Endocarditis

Endocarditis is an inflammation of the endocardium (inner lining of the heart). Diagnosis is according to modified Duke criteria, using evidence from microbiological results, echocardiogram and clinical signs.

Cardiomyopathy

Structural and functional disorders of myocardium commonly diagnosed by echocardiography. Can be primary (genetic) or secondary (e.g. following myocardial infarction).

. Physician diagnosis,

Congestive heart failure

Is defined as failure of the heart to pump a sufficient amount of blood to meet the needs of the body tissues, resulting in tissue congestion and oedema.

Seizure

Select 'yes' for any seizure regardless of cause (e.g. febrile or due to epilepsy)

Stroke / Cerebrovascular accident

Stroke may be a clinical diagnosis, with or without supportive radiological findings.

Meningitis / Encephalitis

Inflammation of the meninges or the brain parenchyma. Select yes if diagnosed clinically, radiologically or microbiologically.

Bacteremia

Growth of bacteria on a blood culture. Select 'no' if the only bacteria grown were believed to be skin contaminants (e.g. coagulase negative Staphylococci or diphtheroids).

Coagulation disorder / DIC

Abnormal coagulation identified by abnormal prothrombin time or activated partial thromboplastin time. Disseminated intravascular coagulation (DIC; consumption coagulopathy; defibrination syndrome) is defined by thrombocytopenia, prolonged prothrombin time, low fibrinogen, elevated D-dimer and thrombotic microangiopathy.

Pulmonary embolism

Obstruction of pulmonary artery by thrombus, air or fat. Physician diagnosis based on clinical signs, computed tomographic pulmonary angiography and/or ventilation/perfusion scanning.

Deep Vein Thrombosis

Blood clots in deep veins of leg, pelvis or arm. Physician diagnosis based on clinical signs, and/or duplex ultrasonography, d-dimer blood test, contrast venography or magnetic resonance imaging (MRI),

Other thromboembolism (not Pulmonary Embolism or Deep Vein Thrombosis)

Please record any other type of physician diagnosed thromboembolism

MODULE 3: OUTCOME CASE REPORT FORM

TREATMENT: At ANY time during hospitalisation, did the patient receive/undergo:

Any Oxygen therapy? OYES ONO OUnknown If YES, total duration: ________days OUnknown

Maximum O_2 flow volume: O <2 L/min O2-5 L/min O6-10 L/min O11-15 L/min O>15 L/min

Non-invasive ventilation? *(Any)* OYES ONO OUnknown If YES, total duration: ________days OUnknown

Invasive ventilation? *(Any)* OYES ONO OUnknown If YES, total duration: ________days OUnknown

High flow nasal oxygen OYES ONO OUnknown If YES, total duration: ________days OUnknown

Prone Positioning? OYES ONO OUnknown

Inhaled Nitric Oxide? OYES ONO OUnknown

Tracheostomy inserted? OYES ONO OUnknown

Extracorporeal support (ECMO)? OYES ONO OUnknown If YES, total duration: ________days OUnknown

Renal replacement therapy (RRT) or dialysis? OYES ONO OUnknown

Inotropes/vasopressors? OYES ONO OUnknown

ICU or High Dependency Unit admission? OYES ONO OUnknown If YES, total duration: ________days OUnknown

If YES, date of ICU admission: [_D_][_D_]/[_M_][_M_]/[_2_][_0_][_Y_][_Y_] OUnknown

date of ICU discharge: [_D_][_D_]/[_M_][_M_]/[_2_][_0_][_Y_][_Y_] OUnknown

COMPLICATIONS: At any time during hospitalisation did the patient experience: *(Unk = Unknown)*

Viral pneumonia/pneumonitis	OYES ONO OUnk	Meningitis / Encephalitis	OYES ONO OUnk
Bacterial pneumonia	OYES ONO OUnk	Bacteremia	OYES ONO OUnk
Acute Respiratory Distress Syndrome	OYES ONO OUnk	Coagulation disorder / DIC	OYES ONO OUnk
Pneumothorax	OYES ONO OUnk	Pulmonary Embolism	OYES ONO OUnk
Pleural effusion	OYES ONO OUnk	Deep Vein Thrombosis	OYES ONO OUnk
Cryptogenic organizing pneumonia (COP)	OYES ONO OUnk	Other thromboembolism (not PE or DVT)	OYES ONO OUnk
Bronchiolitis	OYES ONO OUnk	Anemia	OYES ONO OUnk
Cardiac arrest	OYES ONO OUnk	Rhabdomyolysis / Myositis	OYES ONO OUnk
Myocardial infarction	OYES ONO OUnk	Acute renal injury/ Acute renal failure	OYES ONO OUnk
Cardiac ischaemia	OYES ONO OUnk	Gastrointestinal haemorrhage	OYES ONO OUnk
Cardiac arrhythmia	OYES ONO OUnk	Pancreatitis	OYES ONO OUnk
Myocarditis / Pericarditis	OYES ONO OUnk	Liver dysfunction	OYES ONO OUnk
Endocarditis	OYES ONO OUnk	Hyperglycemia	OYES ONO OUnk
Cardiomyopathy	OYES ONO OUnk	Hypoglycemia	OYES ONO OUnk
Congestive heart failure	OYES ONO OUnk	Other	OYES ONO OUnk
Seizure	OYES ONO OUnk	If YES, specify:____________________	
Stroke / Cerebrovascular accident	OYES ONO OUnk		

Anemia

Select 'yes' if haemoglobin levels were lower than age- and sex-specific thresholds listed below

Age or gender group	Haemoglobin threshold (g/L)	Haemoglobin threshold (mmol/l)
Age 6 months to 5 years	110	6.8
Age 5–12 years	115	7.1
Age 12–15 years	120	7.4
Age > 15 years, non-pregnant women	120	7.4
Pregnant women	110	6.8
Age >15 years, men	130	8.1

Rhabdomyolysis / Myositis

Rhabdomyolysis is a syndrome characterised by muscle necrosis and the release of myoglobin into the blood. Muscle biopsy, electromyography, radiological imaging and the presence of myoglobinuria are not required for the diagnosis.

Myositis may be a clinical diagnosis with supporting evidence from laboratory tests e.g. elevated serum creatine kinase; histological confirmation is not required to make the diagnosis. Myositis can occur without progression to rhabdomyolysis.

Acute renal injury/Acute renal failure

Acute renal injury is defined as any of:

- Increase in serum creatinine by ≥0.3 mg/dL (≥26.5 µmol/L) within 48 hours
- Increase in serum creatinine to ≥1.5 times baseline, which is known or presumed to have occurred within the prior 7 days
- Urine volume <0.5 mL/kg/hour for 6 hours

Gastrointestinal haemorrhage

Refers to bleeding originating from any part of the gastrointestinal tract (from the oropharynx to the rectum).

Pancreatitis

Inflammation of the pancreas, diagnosed from clinical, biochemical, radiological or histological evidence.

MODULE 3: OUTCOME CASE REPORT FORM

TREATMENT: At ANY time during hospitalisation, did the patient receive/undergo:

Any Oxygen therapy? OYES ONO OUnknown If YES, total duration: ________days OUnknown

Maximum O₂ flow volume: O <2 L/min O2-5 L/min O6-10 L/min O11-15 L/min O>15 L/min

Non-invasive ventilation? *(Any)* OYES ONO OUnknown If YES, total duration: ________days OUnknown

Invasive ventilation? *(Any)* OYES ONO OUnknown If YES, total duration: ________days OUnknown

High flow nasal oxygen OYES ONO OUnknown If YES, total duration: ________days OUnknown

Prone Positioning? OYES ONO OUnknown

Inhaled Nitric Oxide? OYES ONO OUnknown

Tracheostomy inserted? OYES ONO OUnknown

Extracorporeal support (ECMO)? OYES ONO OUnknown If YES, total duration: ________days OUnknown

Renal replacement therapy (RRT) or dialysis? OYES ONO OUnknown

Inotropes/vasopressors? OYES ONO OUnknown

ICU or High Dependency Unit admission? OYES ONO OUnknown If YES, total duration: ________days OUnknown

If YES, date of ICU admission: [_D_][_D_]/[_M_][_M_]/[_2_][_0_][_Y_][_Y_] OUnknown

date of ICU discharge: [_D_][_D_]/[_M_][_M_]/[_2_][_0_][_Y_][_Y_] OUnknown

COMPLICATIONS: At any time during hospitalisation did the patient experience: *(Unk = Unknown)*

Viral pneumonia/pneumonitis	OYES ONO OUnk	Meningitis / Encephalitis	OYES ONO OUnk
Bacterial pneumonia	OYES ONO OUnk	Bacteremia	OYES ONO OUnk
Acute Respiratory Distress Syndrome	OYES ONO OUnk	Coagulation disorder / DIC	OYES ONO OUnk
Pneumothorax	OYES ONO OUnk	Pulmonary Embolism	OYES ONO OUnk
Pleural effusion	OYES ONO OUnk	Deep Vein Thrombosis	OYES ONO OUnk
Cryptogenic organizing pneumonia (COP)	OYES ONO OUnk	Other thromboembolism (not PE or DVT)	OYES ONO OUnk
Bronchiolitis	OYES ONO OUnk	Anemia	OYES ONO OUnk
Cardiac arrest	OYES ONO OUnk	Rhabdomyolysis / Myositis	OYES ONO OUnk
Myocardial infarction	OYES ONO OUnk	Acute renal injury/ Acute renal failure	OYES ONO OUnk
Cardiac ischaemia	OYES ONO OUnk	Gastrointestinal haemorrhage	OYES ONO OUnk
Cardiac arrhythmia	OYES ONO OUnk	Pancreatitis	OYES ONO OUnk
Myocarditis / Pericarditis	OYES ONO OUnk	Liver dysfunction	OYES ONO OUnk
Endocarditis	OYES ONO OUnk	Hyperglycemia	OYES ONO OUnk
Cardiomyopathy	OYES ONO OUnk	Hypoglycemia	OYES ONO OUnk
Congestive heart failure	OYES ONO OUnk	Other	OYES ONO OUnk
Seizure	OYES ONO OUnk	If YES, specify:______________________________	
Stroke / Cerebrovascular accident	OYES ONO OUnk		

COMPLICATIONS, continued

Liver dysfunction

A finding that indicates abnormal liver function, may refer to any of the following:

- Clinical jaundice
- Hyperbilirubinaemia (blood bilirubin level twice the upper limit of the normal range)
- An increase in alanine transaminase or aspartate transaminase that is twice the upper limit of the normal range

Hyperglycaemia

For adults, is defined as an abnormally high level of glucose in the blood, blood glucose level that is consistently above 126mg/dL or 7 mmol/L. For children, is defined as a blood glucose level consistently above 8.3 mmol/L.

Hypoglycaemia

For adults, is defined as an abnormally low level of glucose in the blood, a blood glucose level that is consistently below 70mg/dL or 4 mmol/L. For children, is defined as a blood glucose level below 3 mmol/L.

Other

Please specify other complications in the space provided.

MODULE 3: OUTCOME CASE REPORT FORM

TREATMENT: At ANY time during hospitalisation, did the patient receive/undergo:

Any Oxygen therapy? OYES ONO OUnknown If YES, total duration: ________days OUnknown

Maximum O_2 flow volume: O <2 L/min O2-5 L/min O6-10 L/min O11-15 L/min O>15 L/min

Non-invasive ventilation? *(Any)* OYES ONO OUnknown If YES, total duration: ________days OUnknown

Invasive ventilation? *(Any)* OYES ONO OUnknown If YES, total duration: ________days OUnknown

High flow nasal oxygen OYES ONO OUnknown If YES, total duration: ________days OUnknown

Prone Positioning? OYES ONO OUnknown

Inhaled Nitric Oxide? OYES ONO OUnknown

Tracheostomy inserted? OYES ONO OUnknown

Extracorporeal support (ECMO)? OYES ONO OUnknown If YES, total duration: ________days OUnknown

Renal replacement therapy (RRT) or dialysis? OYES ONO OUnknown

Inotropes/vasopressors? OYES ONO OUnknown

ICU or High Dependency Unit admission? OYES ONO OUnknown If YES, total duration: ________days OUnknown

If YES, date of ICU admission: [_D_][_D_]/[_M_][_M_]/[_2_][_0_][_Y_][_Y_] OUnknown

date of ICU discharge: [_D_][_D_]/[_M_][_M_]/[_2_][_0_][_Y_][_Y_] OUnknown

COMPLICATIONS: At any time during hospitalisation did the patient experience: *(Unk = Unknown)*

Viral pneumonia/pneumonitis	OYES ONO OUnk	Meningitis / Encephalitis	OYES ONO OUnk
Bacterial pneumonia	OYES ONO OUnk	Bacteremia	OYES ONO OUnk
Acute Respiratory Distress Syndrome	OYES ONO OUnk	Coagulation disorder / DIC	OYES ONO OUnk
Pneumothorax	OYES ONO OUnk	Pulmonary Embolism	OYES ONO OUnk
Pleural effusion	OYES ONO OUnk	Deep Vein Thrombosis	OYES ONO OUnk
Cryptogenic organizing pneumonia (COP)	OYES ONO OUnk	Other thromboembolism (not PE or DVT)	OYES ONO OUnk
Bronchiolitis	OYES ONO OUnk	Anemia	OYES ONO OUnk
Cardiac arrest	OYES ONO OUnk	Rhabdomyolysis / Myositis	OYES ONO OUnk
Myocardial infarction	OYES ONO OUnk	Acute renal injury/ Acute renal failure	OYES ONO OUnk
Cardiac ischaemia	OYES ONO OUnk	Gastrointestinal haemorrhage	OYES ONO OUnk
Cardiac arrhythmia	OYES ONO OUnk	Pancreatitis	OYES ONO OUnk
Myocarditis / Pericarditis	OYES ONO OUnk	Liver dysfunction	OYES ONO OUnk
Endocarditis	OYES ONO OUnk	Hyperglycemia	OYES ONO OUnk
Cardiomyopathy	OYES ONO OUnk	Hypoglycemia	OYES ONO OUnk
Congestive heart failure	OYES ONO OUnk	Other	OYES ONO OUnk
Seizure	OYES ONO OUnk	If YES, specify:________________	
Stroke / Cerebrovascular accident	OYES ONO OUnk		

DIAGNOSTICS

Radiology

Chest X-Ray/ CT performed?
Record if X-ray and/or CT were performed, even if no infiltrates were present.

Pathogen Testing Details

Details of pathogen testing per biospecimen type
If the patient had samples taken for pathogen detection testing during their hospital stay, please complete a row for every type of sample collected (e.g. nasal/NP swab, sputum, etc.).

Where both positive and negative results for a particular sample type exist (from samples taken at different time points during the patient's hospital stay) please record the earliest positive result.

If results are indeterminate' or considered by the clinical team to represent contamination/colonisation, record on the form as Negative

If only multiple negative results exist for a particular sample type (from samples taken at different time points during the patient's hospital stay), please document the earliest negative result.

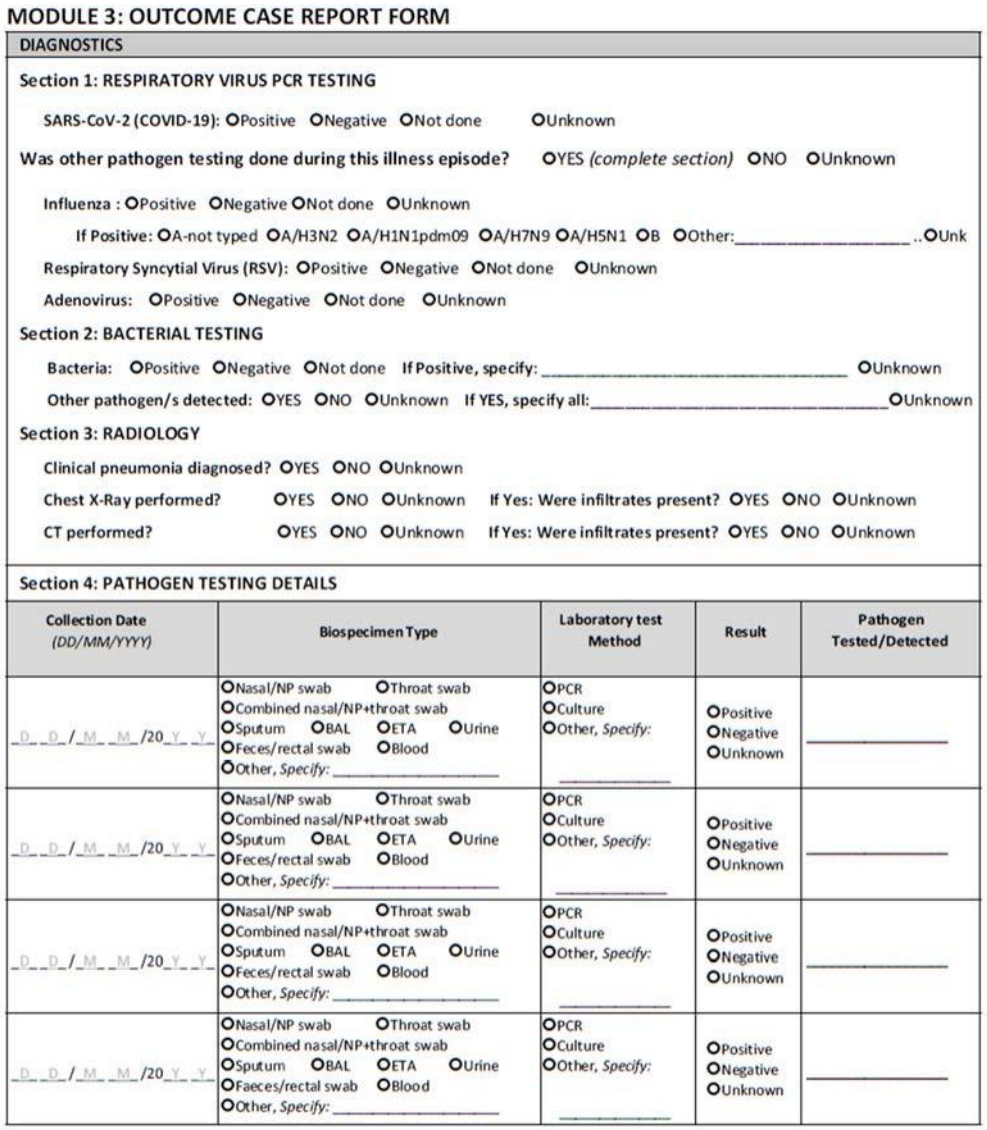

MODULE 3: OUTCOME CASE REPORT FORM

DIAGNOSTICS

Section 1: RESPIRATORY VIRUS PCR TESTING

SARS-CoV-2 (COVID-19): OPositive ONegative ONot done OUnknown

Was other pathogen testing done during this illness episode? OYES *(complete section)* ONO OUnknown

Influenza : OPositive ONegative ONot done OUnknown

If Positive: OA-not typed OA/H3N2 OA/H1N1pdm09 OA/H7N9 OA/H5N1 OB OOther:__________..OUnk

Respiratory Syncytial Virus (RSV): OPositive ONegative ONot done OUnknown

Adenovirus: OPositive ONegative ONot done OUnknown

Section 2: BACTERIAL TESTING

Bacteria: OPositive ONegative ONot done If Positive, specify: __________ OUnknown

Other pathogen/s detected: OYES ONO OUnknown If YES, specify all:__________OUnknown

Section 3: RADIOLOGY

Clinical pneumonia diagnosed? OYES ONO OUnknown

Chest X-Ray performed? OYES ONO OUnknown If Yes: Were infiltrates present? OYES ONO OUnknown

CT performed? OYES ONO OUnknown If Yes: Were infiltrates present? OYES ONO OUnknown

Section 4: PATHOGEN TESTING DETAILS

Collection Date *(DD/MM/YYYY)*	Biospecimen Type	Laboratory test Method	Result	Pathogen Tested/Detected
_D_D_/_M_M_/20_Y_Y_	ONasal/NP swab OThroat swab OCombined nasal/NP+throat swab OSputum OBAL OETA OUrine OFeces/rectal swab OBlood OOther, *Specify:* ______	OPCR OCulture OOther, *Specify:* ______	OPositive ONegative OUnknown	______
_D_D_/_M_M_/20_Y_Y_	ONasal/NP swab OThroat swab OCombined nasal/NP+throat swab OSputum OBAL OETA OUrine OFeces/rectal swab OBlood OOther, *Specify:* ______	OPCR OCulture OOther, *Specify:* ______	OPositive ONegative OUnknown	______
_D_D_/_M_M_/20_Y_Y_	ONasal/NP swab OThroat swab OCombined nasal/NP+throat swab OSputum OBAL OETA OUrine OFeces/rectal swab OBlood OOther, *Specify:* ______	OPCR OCulture OOther, *Specify:* ______	OPositive ONegative OUnknown	______
_D_D_/_M_M_/20_Y_Y_	ONasal/NP swab OThroat swab OCombined nasal/NP+throat swab OSputum OBAL OETA OUrine OFaeces/rectal swab OBlood OOther, *Specify:* ______	OPCR OCulture OOther, *Specify:* ______	OPositive ONegative OUnknown	______

MEDICATION - While hospitalised or at discharge, were any of the following administered?

Antiviral or COVID-19 targeted agent

Record all antivirals or COVID-19 targeted agents administered from date of admission or during the hospitalisation. Record the total number of days the treatment was given.

Additional space is available under 'Other treatments…' at the end of this section if required

Antibiotic

'Antibiotic' refers to any agent(s) are substances naturally produced by microorganisms or their derivatives that selectively target microorganisms. These substances are used in the treatment of bacterial and other microbial infections. Topical preparations are not included.

Corticosteroid

'Corticosteroids' (commonly referred to as 'steroids') refers to all types of therapeutic corticosteroid, made in the adrenal cortex (the outer part of the adrenal gland). They are also made in the laboratory. Examples include: prednisolone, prednisone, methyl-prednisolone, dexamethasone, hydrocortisone, fluticasone, betamethasone (note that other examples exist). Topical preparations are not included, but inhaled preparations are included. The indication for administering corticosteroids does not need to be directly related to the treatment of COVID-19.

MODULE 3: OUTCOME CASE REPORT FORM

MEDICATION: While hospitalised or at discharge, were any of the following administered? *(Unk=Unknown)*

ANTIVIRAL OR COVID-19 TARGETED AGENT? ○YES ○NO ○Unknown   If YES, specify (all) :

☐ Ribavirin   Date commenced[_D_][_D_]/[_M_][_M_]/[_2_][_0_][_Y_][_Y_]   ○Unk   Duration: _______ days ○Unk

☐ Lopinavir/Ritonavir   Date commenced [_D_][_D_]/[_M_][_M_]/[_2_][_0_][_Y_][_Y_] ○Unk   Duration: _______ days ○Unk

☐ Remdesivir (Veklury)   Date commenced [_D_][_D_]/[_M_][_M_]/[_2_][_0_][_Y_][_Y_] ○Unk   Duration: _______ days ○Unk

☐ Interferon alpha   Date commenced [_D_][_D_]/[_M_][_M_]/[_2_][_0_][_Y_][_Y_] ○Unk   Duration: _______ days ○Unk

☐ Interferon beta   Date commenced [_D_][_D_]/[_M_][_M_]/[_2_][_0_][_Y_][_Y_] ○Unk   Duration: _______ days ○Unk

☐ Chloroquine/hydroxychloroquine:

Date commenced [_D_][_D_]/[_M_][_M_]/[_2_][_0_][_Y_][_Y_] ○Unk...   Duration: _______days ○Unk

☐ Interleukin-6 (IL-6) inhibitor   IF YES which: ☐ Tocilizumab ☐ Sarilumab ☐ Other IL-6 inhibitor_____________________○Unk

Date commenced [_D_][_D_]/[_M_][_M_]/[_2_][_0_][_Y_][_Y_] ○Unk...   Duration: _______days ○Unk

☐ Convalescent plasma   Date commenced [_D_][_D_]/[_M_][_M_]/[_2_][_0_][_Y_][_Y_] ○Unk   Duration: _______days ○Unk

☐ Anti-influenza anti-viral   IF YES which: ☐Oseltamivir (Tamiflu®)   ☐ Zanamivir ○Unk

Date commenced [_D_][_D_]/[_M_][_M_]/[_2_][_0_][_Y_][_Y_] ○Unk...   Duration: _______days ○Unk

☐ Other_________________ Date commenced [_D_][_D_]/[_M_][_M_]/[_2_][_0_][_Y_][_Y_] ○Unk   Duration: _______days ○Unk

ANTIBIOTIC? ○YES ○NO ○Unknown If yes, specify all:

Agent 1:_____________________ Date commenced [_D_][_D_]/[_M_][_M_]/[_2_][_0_][_Y_][_Y_] Duration: ____ days ○Unk

Agent 2:_____________________ Date commenced [_D_][_D_]/[_M_][_M_]/[_2_][_0_][_Y_][_Y_] Duration: ____ days ○Unk

Agent 3:_____________________ Date commenced [_D_][_D_]/[_M_][_M_]/[_2_][_0_][_Y_][_Y_] Duration: ____ days ○Unk

CORTICOSTEROID?   ○YES ○NO ○Unknown

If YES: Dexamethasone? ○YES ○NO ○Unknown

If YES, check all that apply:

☐ 6mg once per day (od)? ○YES ○NO ○Unknown If YES, Route: ☐ Oral ☐ Intravenous ○Unk

If YES, Date commenced [_D_][_D_]/[_M_][_M_]/[_2_][_0_][_Y_][_Y_] Duration: ______ days ○Unk

☐ other dose or frequency? ○YES ○NO ○Unknown If YES, Route: ☐ Oral ☐ Intravenous ○Unk

If YES, Date commenced [_D_][_D_]/[_M_][_M_]/[_2_][_0_][_Y_][_Y_] Duration: ______ days ○Unk

If YES: Other corticosteroid? ○YES ○NO ○Unknown

If YES: Which steroid: ☐ Prednisolone ☐ Hydrocortisone ☐ Methylprednisolone ☐ Other

Route: ☐ Oral ☐ Intravenous ○Unk

MEDICATION (continued)

Anticoagulants

These include heparin, enoxaparin, apixaban, dabigatran, rivaroxaban, edoxaban, warfarin. For heparin treatment, please specify if unfractionated or low molecular weight heparin was administered.

Antifungal Agent

'Antifungal agent' refers to any agent(s) prescribed specifically to treat systemic or topical infections caused by fungi. Examples include fluconazole, amphotericin, caspofungin, anidulafungin, posaconazole, itraconazole (note that other examples exist). Topical preparations should not be recorded.

Other treatment administered for COVID-19

Record any other medications, experimental or re-purposed, administered to modify the course of COVID-19 during the admission (including as part of a clinical trial). This could include convalescent plasma, immuno-modulatory agents and anti-viral agents not already recorded above.

MODULE 3: OUTCOME CASE REPORT FORM

MEDICATION (continued):

ANTICOAGULATION? OYES ONO OUnk

If YES: Agent: ____________________

Route: ☐ Subcutaneous ☐ Intravenous (IV) OUnk

Indication: ☐ therapeutic (treatment of DVT/PE) ☐ enhanced prophylaxis for COVID-19 ☐ routine inpatient prophylaxis ☐ Unk

ANTIFUNGAL AGENT? OYES ONO OUnk

OTHER treatments administered for COVID-19 including experimental or compassionate use? OYES ONO OUnk

If YES, specify agent and timing of administration:

Agent 1: ____________________

Date commenced [_D_][_D_]/[_M_][_M_]/[_2_][_0_][_Y_][_Y_] OUnk Duration: _______ days OUnk

Agent 2: ____________________

Date commenced [_D_][_D_]/[_M_][_M_]/[_2_][_0_][_Y_][_Y_] OUnk Duration: _______ days OUnk

Agent 3: ____________________

Date commenced [_D_][_D_]/[_M_][_M_]/[_2_][_0_][_Y_][_Y_] OUnk Duration: _______ days OUnk

OUTCOME

Was patient diagnosed with Covid-19? OYES ONO OUnknown

If yes, was the diagnosis based on: Olaboratory confirmation O clinical assessment

Outcome: ODischarged alive OHospitalised OTransfer to other facility ODeath OPalliative discharge OUnknown

Outcome date: [_D_][_D_]/[_M_][_M_]/[_2_][_0_][_Y_][_Y_] OUnknown

If alive at outcome date:

Ability to self-care at discharge versus before illness: OSame as before illness OWorse OBetter OUnknown

Post-discharge treatment: Oxygen therapy? OYES ONO OUnknown

Ongoing health care needs relating to this admission for COVID-19: OYES ONO OUnknown

Ongoing health care needs NOT related to COVID episode: OYES ONO OUnknown

Medically fit for discharge (COVID-19 resolved) but remains in hospital for other reason (e.g. awaiting suitable care in community, resident in long term health care or mental health facility): OYES ONO OUnknown

OUTCOME

Was patient diagnosed with Covid-19?
Please confirm method of diagnosis, confirming diagnosis by clinical assessment only if no positive laboratory result was obtained.

Discharged alive can mean discharge to their usual place of residence before their illness, to the home of a relative or friend, or to a social care facility, because their illness is no longer severe enough to warrant treatment in a medical facility.

Hospitalized means they are still in hospital but have recovered from COVID-19 infection and the form has been completed as the patient is in a part of the hospital for care of other conditions and where the form will not be completed at a later date.

Transfer to other facility means they have been transferred to another facility that provides medical care. This could be a specialist centre for more intensive treatment or a step-down for rehabilitation. It does not include facilities that solely provide social care (these patients should be listed as discharged alive).

Death means the patient died in the hospital.

Palliative discharge means the patient has been discharged with the expectation that they will not recover from this or other co-existing illness. This could be to a specialist hospice facility, or to their usual home address with anticipatory end of life medications.

Outcome date Please state the date for the outcome listed above.

If Discharged Alive: (answer these questions only if outcome is 'Discharged Alive'

Ability to self-care at discharge versus before illness: the patient is able to care for themselves at discharge (in terms of activities of daily living) at the same level as before they developed illness then tick 'same as before illness'. If their ability to self-care has decreased or increased, then tick the appropriate circle ('worse' or 'better').

Post-discharge treatment

Oxygen therapy includes, NIV or home ventilation (respiratory support/treatment).

MODULE 3: OUTCOME CASE REPORT FORM

MEDICATION (continued):

ANTICOAGULATION? OYES ONO OUnk

If YES: Agent: ______________________

Route: ☐ Subcutaneous ☐ Intravenous (IV) OUnk

Indication: ☐ therapeutic (treatment of DVT/PE) ☐ enhanced prophylaxis for COVID-19 ☐ routine inpatient prophylaxis ☐ Unk

ANTIFUNGAL AGENT? OYES ONO OUnk

OTHER treatments administered for COVID-19 including experimental or compassionate use? OYES ONO OUnk

If YES, specify agent and timing of administration:

Agent 1: ______________________

Date commenced [_D_][_D_]/[_M_][_M_]/[_2_][_0_][_Y_][_Y_] OUnk Duration: _______ days OUnk

Agent 2: ______________________

Date commenced [_D_][_D_]/[_M_][_M_]/[_2_][_0_][_Y_][_Y_] OUnk Duration: _______ days OUnk

Agent 3: ______________________

Date commenced [_D_][_D_]/[_M_][_M_]/[_2_][_0_][_Y_][_Y_] OUnk Duration: _______ days OUnk

OUTCOME

Was patient diagnosed with Covid-19? OYES ONO OUnknown

If yes, was the diagnosis based on: Olaboratory confirmation O clinical assessment

Outcome: ODischarged alive OHospitalised OTransfer to other facility ODeath OPalliative discharge OUnknown

Outcome date: [_D_][_D_]/[_M_][_M_]/[_2_][_0_][_Y_][_Y_] OUnknown

If alive at outcome date:

Ability to self-care at discharge versus before illness: OSame as before illness OWorse OBetter OUnknown

Post-discharge treatment: Oxygen therapy? OYES ONO OUnknown

Ongoing health care needs relating to this admission for COVID-19: OYES ONO OUnknown

Ongoing health care needs NOT related to COVID episode: OYES ONO OUnknown

Medically fit for discharge (COVID-19 resolved) but remains in hospital for other reason (e.g. awaiting suitable care in community, resident in long term health care or mental health facility): OYES ONO OUnknown

CORE CRITICAL CARE MODULE

Complete this form for anyone receiving critical care regardless of type of ward, in addition to the CORE COVID-19 CRF.

Admission date: this is the date the patient was admitted to the critical care ward.

Interventional clinical study: this could be a trial of a therapeutic agent (e.g. anti-viral, immunomodulator, convalescent plasma) or supportive intervention (e.g. high flow oxygen).

Reason for admission: these are the diagnoses/complications that required critical care management as assessed by a physician select all that apply.

Clinical Frailty Scale: see last page

Severity scores:
Complete if assessed or score recorded in the medical notes.

PELOD score: see https://sfar.org/scores2/pelod2.php

PRISM III score: see https://www.cpccrn.org/calculators/prismiiicalculator/

Fluid balance: net fluid balance over 24h assessment day or prior to assessment

Nutrition: select route of the main type of nutrition on day of assessment from parenteral, enteral (including nasogastric or gastrostomy/jejunostomy), or NPO (*nil per os* – no oral intake).

Physical mobility: score from options 0 to 10, record **best** score.

CRITICAL CARE MODULE PART B

ICU/HDU ADMISSION FORM

ICU ADMISSION DATE *(DD/MM/YYYY)*: [_D_][_D_]/[_M_][_M_]/[_2_][_0_][_Y_][_Y_]

Enrolment in interventional clinical study? ○YES ○NO ○Unknown **If YES,** name of study:____________________ or

Treatment/s trialled:____

____ ○Unknown

Reason for ICU admission (tick all that apply): ☐Respiratory failure ☐Septic shock ☐Venous thromboembolism ☐Cardiovascular complications ☐Acute kidney injury ☐Acute liver injury ☐Neurological complications ☐Secondary infection ☐Pancreatic injury ☐Disseminated intravascular coagulation ☐Pregnancy related complications ☐Rhabdomyolysis ☐OTHER (please specify) ____________________ ○Unknown

Clinical Frailty Score (CFS/9) [______] ○Unknown **Acute renal failure?** ○YES ○NO ○Unknown

DAILY FORM (Complete daily for duration of ICU/ITU/IMC/HDU admission)
(between 00:00 to 24:00 on day of assessment) Record the 'worst' value on the day of assessment.

IF patient is <18 years: PELOD Total Score [______] ○Unknown **PRISM III score:** [______] ○Unknown

Fluid balance (in last 24 hours) (mL) ____________ ☐Unknown

Nutrition ○Parenteral ○Enteral ○NPO ○Unknown **Best physical mobility** [___]**/10** (*see scoring below*) ○Unknown

0 Passively moved by staff (incl. passive cycling only)
1 Any activity in bed, but not moving out of or over edge of bed (incl. cycling)
2 Passively moved to chair (no standing or sitting at edge of bed)
3 Actively sitting over side of bed with some trunk control (may be assisted)
4 Standing
5 Transferring from bed to chair
6 Marching on the spot (at bedside; > 2steps/foot)
7 Walking with assistance of 2 or more people (>5m)
8 Walking with assistance of 1 person (>5m)
9 Walking independently with gait aid (>5m)
10 Walking independently without gait aid (>5m)

Licensed under a Creative Commons Attribution-Share Alike 4.0 International License by ISARIC on behalf of Oxford University.

Type of ventilation:
Record all types of ventilation received on day of assessment on or after admission to the critical care ward (ICU/HDU).
Abbreviations:
ETT: endotracheal tube
BIPAP: bi-level positive airway pressure
CPAP: continuous positive airway pressure
CRRT: continuous renal replacement therapy
IHD: intermittent haemodialysis
SLED: sustained low efficiency dialysis

For modes of ventilation (invasive, non-invasive, humidified high flow nasal cannula) please select all modes the patient received during the 24 hour assessment day.
Modes of mechanical ventilation:

- Synchronized Intermittent Mandatory Ventilation – Volume-Controlled (SIMV-V)
- Synchronized Intermittent Mandatory Ventilation – Pressure-Controlled (SIMV-P)
- Volume Controlled Ventilation
- Pressure Controlled Ventilation
- Pressure Regulated Volume Control (PRVC)
- Airway Pressure Release Ventilation (APRV)
- Pressure Support Ventilation (PSV)
- Volume Support Ventilation (VSV)
- High Frequency Oscillatory (HFO)
- Bilevel Positive Airway Pressure (BiPAP)
- Continuous Positive Airway Pressure (CPAP)
- Proportional Assist Ventilation (PAV)
- Neurally Adjusted Ventilatory Assist (NAVA)

Record highest tidal volume and airway pressures.

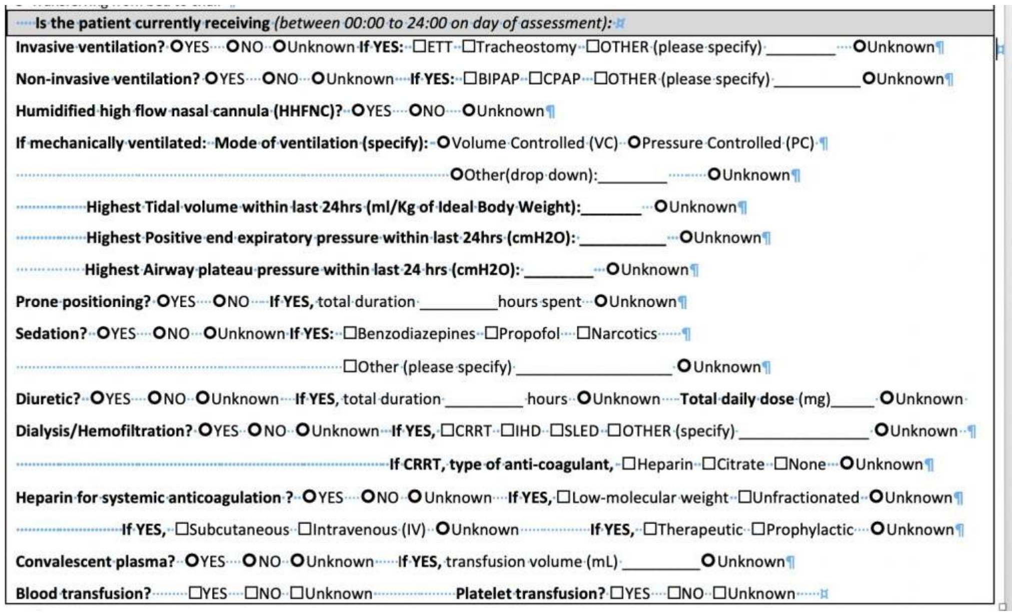
Is the patient currently receiving *(between 00:00 to 24:00 on day of assessment):*
Invasive ventilation? ○YES ○NO ○Unknown **If YES:** ☐ETT ☐Tracheostomy ☐OTHER (please specify) ________ ○Unknown
Non-invasive ventilation? ○YES ○NO ○Unknown **If YES:** ☐BIPAP ☐CPAP ☐OTHER (please specify) __________ ○Unknown
Humidified high flow nasal cannula (HHFNC)? ○YES ○NO ○Unknown
If mechanically ventilated: Mode of ventilation (specify): ○Volume Controlled (VC) ○Pressure Controlled (PC)
○Other(drop down):________ ○Unknown
Highest Tidal volume within last 24hrs (ml/Kg of Ideal Body Weight):_______ ○Unknown
Highest Positive end expiratory pressure within last 24hrs (cmH2O): __________ ○Unknown
Highest Airway plateau pressure within last 24 hrs (cmH2O): ________ ○Unknown
Prone positioning? ○YES ○NO **If YES,** total duration _________hours spent ○Unknown
Sedation? ○YES ○NO ○Unknown **If YES:** ☐Benzodiazepines ☐Propofol ☐Narcotics
☐Other (please specify) __________________ ○Unknown
Diuretic? ○YES ○NO ○Unknown **If YES,** total duration _________ hours ○Unknown **Total daily dose** (mg)_____ ○Unknown
Dialysis/Hemofiltration? ○YES ○NO ○Unknown **If YES,** ☐CRRT ☐IHD ☐SLED ☐OTHER (specify) _______________ ○Unknown
If CRRT, type of anti-coagulant, ☐Heparin ☐Citrate ☐None ○Unknown
Heparin for systemic anticoagulation ? ○YES ○NO ○Unknown **If YES,** ☐Low-molecular weight ☐Unfractionated ○Unknown
If YES, ☐Subcutaneous ☐Intravenous (IV) ○Unknown **If YES,** ☐Therapeutic ☐Prophylactic ○Unknown
Convalescent plasma? ○YES ○NO ○Unknown **If YES,** transfusion volume (mL) _________ ○Unknown
Blood transfusion? ☐YES ☐NO ☐Unknown **Platelet transfusion?** ☐YES ☐NO ☐Unknown

Licensed under a Creative Commons Attribution-Share Alike 4.0 International License by ISARIC on behalf of Oxford University.

Clinical Frailty Scale*

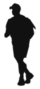

1 Very Fit – People who are robust, active, energetic and motivated. These people commonly exercise regularly. They are among the fittest for their age.

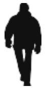

2 Well – People who have **no active disease symptoms** but are less fit than category 1. Often, they exercise or are very **active occasionally**, e.g. seasonally.

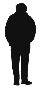

3 Managing Well – People whose **medical problems are well controlled,** but are **not regularly active** beyond routine walking.

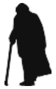

4 Vulnerable – While **not dependent** on others for daily help, often **symptoms limit activities.** A common complaint is being "slowed up", and/or being tired during the day.

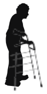

5 Mildly Frail – These people often have **more evident slowing,** and need help in **high order IADLs** (finances, transportation, heavy housework, medications). Typically, mild frailty progressively impairs shopping and walking outside alone, meal preparation and housework.

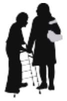

6 Moderately Frail – People need help with **all outside activities** and with **keeping house**. Inside, they often have problems with stairs and need **help with bathing** and might need minimal assistance (cuing, standby) with dressing.

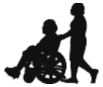

7 Severely Frail – **Completely dependent for personal care**, from whatever cause (physical or cognitive). Even so, they seem stable and not at high risk of dying (within ~ 6 months).

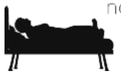

8 Very Severely Frail – Completely dependent, approaching the end of life. Typically, they could not recover even from a minor illness.

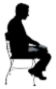

9. Terminally Ill - Approaching the end of life. This category applies to people with **a life expectancy <6 months**, who are **not otherwise evidently frail.**

Scoring frailty in people with dementia

The degree of frailty corresponds to the degree of dementia. Common **symptoms in mild dementia** include forgetting the details of a recent event, though still remembering the event itself, repeating the same question/story and social withdrawal.

In **moderate dementia**, recent memory is very impaired, even though they seemingly can remember their past life events well. They can do personal care with prompting.

In **severe dementia**, they cannot do personal care without help.

Licensed under a Creative Commons Attribution-Share Alike 4.0 International License by ISARIC on behalf of Oxford University.
